# Installation of C_4_ photosynthetic pathway enzymes in rice using a single construct

**DOI:** 10.1111/pbi.13487

**Published:** 2020-10-27

**Authors:** Maria Ermakova, Stéphanie Arrivault, Rita Giuliani, Florence Danila, Hugo Alonso‐Cantabrana, Daniela Vlad, Hirofumi Ishihara, Regina Feil, Manuela Guenther, Gian Luca Borghi, Sarah Covshoff, Martha Ludwig, Asaph B. Cousins, Jane A. Langdale, Steven Kelly, John E. Lunn, Mark Stitt, Susanne von Caemmerer, Robert T. Furbank

**Affiliations:** ^1^ Australian Research Council Centre of Excellence for Translational Photosynthesis Division of Plant Science Research School of Biology The Australian National University Acton ACT Australia; ^2^ Max Planck Institute of Molecular Plant Physiology Potsdam‐Golm Germany; ^3^ School of Biological Sciences Molecular Plant Sciences Washington State University Pullman WA USA; ^4^ Grains Research and Development Corporation Barton ACT Australia; ^5^ Department of Plant Sciences University of Oxford Oxford UK; ^6^ Department of Plant Sciences University of Cambridge Cambridge UK; ^7^ School of Molecular Sciences The University of Western Australia Crawley WA Australia

**Keywords:** C_4_ photosynthesis, rice, metabolic engineering

## Abstract

Introduction of a C_4_ photosynthetic mechanism into C_3_ crops offers an opportunity to improve photosynthetic efficiency, biomass and yield in addition to potentially improving nitrogen and water use efficiency. To create a two‐cell metabolic prototype for an NADP‐malic enzyme type C_4_ rice, we transformed *Oryza sativa* spp. *japonica* cultivar Kitaake with a single construct containing the coding regions of carbonic anhydrase, phospho*enol*pyruvate (PEP) carboxylase, NADP‐malate dehydrogenase, pyruvate orthophosphate dikinase and NADP‐malic enzyme from *Zea mays*, driven by cell‐preferential promoters. Gene expression, protein accumulation and enzyme activity were confirmed for all five transgenes, and intercellular localization of proteins was analysed. ^13^CO_2_ labelling demonstrated a 10‐fold increase in flux though PEP carboxylase, exceeding the increase in measured *in vitro* enzyme activity, and estimated to be about 2% of the maize photosynthetic flux. Flux from malate via pyruvate to PEP remained low, commensurate with the low NADP‐malic enzyme activity observed in the transgenic lines. Physiological perturbations were minor and RNA sequencing revealed no substantive effects of transgene expression on other endogenous rice transcripts associated with photosynthesis. These results provide promise that, with enhanced levels of the C_4_ proteins introduced thus far, a functional C_4_ pathway is achievable in rice.

## Introduction

Installation of a C_4_ photosynthetic pathway in rice has been predicted to increase rice yields by up to 50% (Hibberd *et al*., 2008) and has been the focus of a large international consortium for more than a decade (https://c4rice.com/; von Caemmerer *et al*., 2012; Ermakova *et al*., 2020). Whereas a full compendium of genes required to engineer anatomical specialization for a full two‐cell C_4_ pathway in rice is still a way off, the genes and promoter sequences required for a metabolic prototype are currently available (Ermakova *et al*., 2020). An obstacle thus far in developing a metabolic prototype has been the difficulty of genotype engineering wherein multiple genes originating from distinct single gene transgenics are crossed to assemble a complete metabolic pathway (Karki *et al*., 2020). This has required screening of thousands of individuals across the initial transgenic generation stage and through multiple rounds of crosses to track the presence of all transgenes and confirm their expression in the appropriate cell‐type in segregating material (Karki *et al*., 2020; Lin *et al*., 2020). Modular cloning technologies such as Golden Gate (Engler *et al*., 2014) offer the opportunity to introduce a full suite of C_4_ enzymes into rice on a single construct, simplifying generation of lines and minimizing the potential for deleterious effects arising from transgene insertion.

Introduction of full C_4_ biochemistry into a C_3_ plant will require significant changes to chloroplast proteomes of mesophyll (M) and bundle sheath (BS) cells (Hernández‐Prieto *et al*., 2019; Majeran and van Wijk, 2009), but a minimal C_4_ cycle could be built by introducing just five enzymes from *Zea mays* (maize) into specific cells and compartments of the rice leaf (Ermakova *et al*., 2020; Karki *et al*., 2020). In this scenario, carbonic anhydrase (CA) and PEP carboxylase (PEPC) in the cytosol of M cells convert CO_2_ into bicarbonate and fix it into oxaloacetate. NADP‐malate dehydrogenase (MDH) inside the mesophyll chloroplasts then converts oxaloacetate into malate using NADPH produced by photosynthetic electron transport. After malate diffuses into BS cells, it is decarboxylated inside the chloroplast by NADP‐dependent malic enzyme (NADP‐ME). The released CO_2_ is refixed by ribulose‐1,5‐bisphosphate carboxylase oxygenase (Rubisco) and the residual pyruvate diffuses back to M cells to be regenerated into PEP by pyruvate orthophosphate dikinase (PPDK) inside the chloroplast. Individually, expression of these enzymes does not affect plant fitness or photosynthetic function (Karki *et al*., 2020). However, collectively this minimal cycle would contribute to establishing higher CO_2_ partial pressure around Rubisco in BS cells and could be beneficial even in plants with C_3_ leaf anatomy (Ermakova *et al*., 2020).

Previous attempts to introduce C_4_ metabolism into C_3_ species were focused primarily on using maize enzymes to replicate the single cell type C_4_ pathway that is found in species such as *Hydrilla verticillata* (Miyao *et al*., 2011). Stacking of four maize genes was achieved by crossing individual rice lines transformed with single genes of interest or by multiple re‐transformation of transgenic lines with additional gene constructs (Taniguchi *et al*., 2008). Although extractable activity of C_4_ cycle enzymes was unambiguously demonstrated *in vitro* for both single and multigene rice transgenics (Fukayama *et al*., 2001; Karki *et al*., 2020; Ku *et al*., 1999; Taniguchi *et al*., 2008; Tsuchida *et al*., 2001), expression of maize PEPC did not result in any ^14^C incorporation into C_4_ acids *in vivo* (Fukayama *et al*., 2003) and reports confirming C_4_ metabolic function using isotopic labelling (Arrivault *et al*., 2016; Hatch and Slack, 1966) have so far been missing.

Here we express five enzymes required to form a minimal NADP‐ME C_4_ cycle in appropriate cell types with the correct subcellular compartment in rice using a single construct. ^13^CO_2_ labelling and metabolomics revealed *in vivo* incorporation of CO_2_ into C_4_ acids (C_4_‐carboxylation) but no evidence of subsequent decarboxylation, indicating that the C_4_ cycle is partially functional. We show that physiological consequences of C_4_‐carboxylation in rice are minor with limited perturbation to photosynthetic light induction and no substantive effects on the global leaf transcriptome. Our results demonstrate that expression of a multigene construct is feasible in rice and provides a basis for the establishment of a C_4_ cycle in C_3_ species.

## Results

### A single construct for expression of five C_4_ enzyme transgenes

The coding sequences of five *Z. mays* genes encoding core enzymes of the C_4_ cycle were assembled in a single construct using the Golden Gate cloning system (see Materials and Methods; Engler *et al*., 2014). *PEPC* promoters from four different C_4_ grass species were used to drive M‐preferential gene expression, and the glycine decarboxylase P‐protein (*GLDP*) promoter from *Flaveria trinervia* was used for BS‐preferential expression (Engelmann *et al*., 2008; Gupta *et al*., 2020). The nucleotide sequence of the baculovirus envelope gp64 protein (AcV5 tag; Lawrence *et al*., 2003) was added as a tag to the N‐terminal end of *ZmCA* to aid in its detection *in planta*. Constructs were transformed into *O. sativa* spp. *japonica* cultivar Kitaake using stable agrobacterium‐mediated transformation and either hygromycin or bialaphos as selective agents. Two hygromycin‐resistant T_0_ plants (lines 1 and 29) and one bialaphos‐resistant T_0_ plant (line B6) were selected for further analysis based on the presence of transcripts from all five *Z. mays* transgenes. Homozygous plants of the three selected lines were identified in the T_1_ progeny, and seeds originating from those plants were used in all further experiments.

### Expression, activity and localization of the C_4_ enzymes

**Figure 1:**
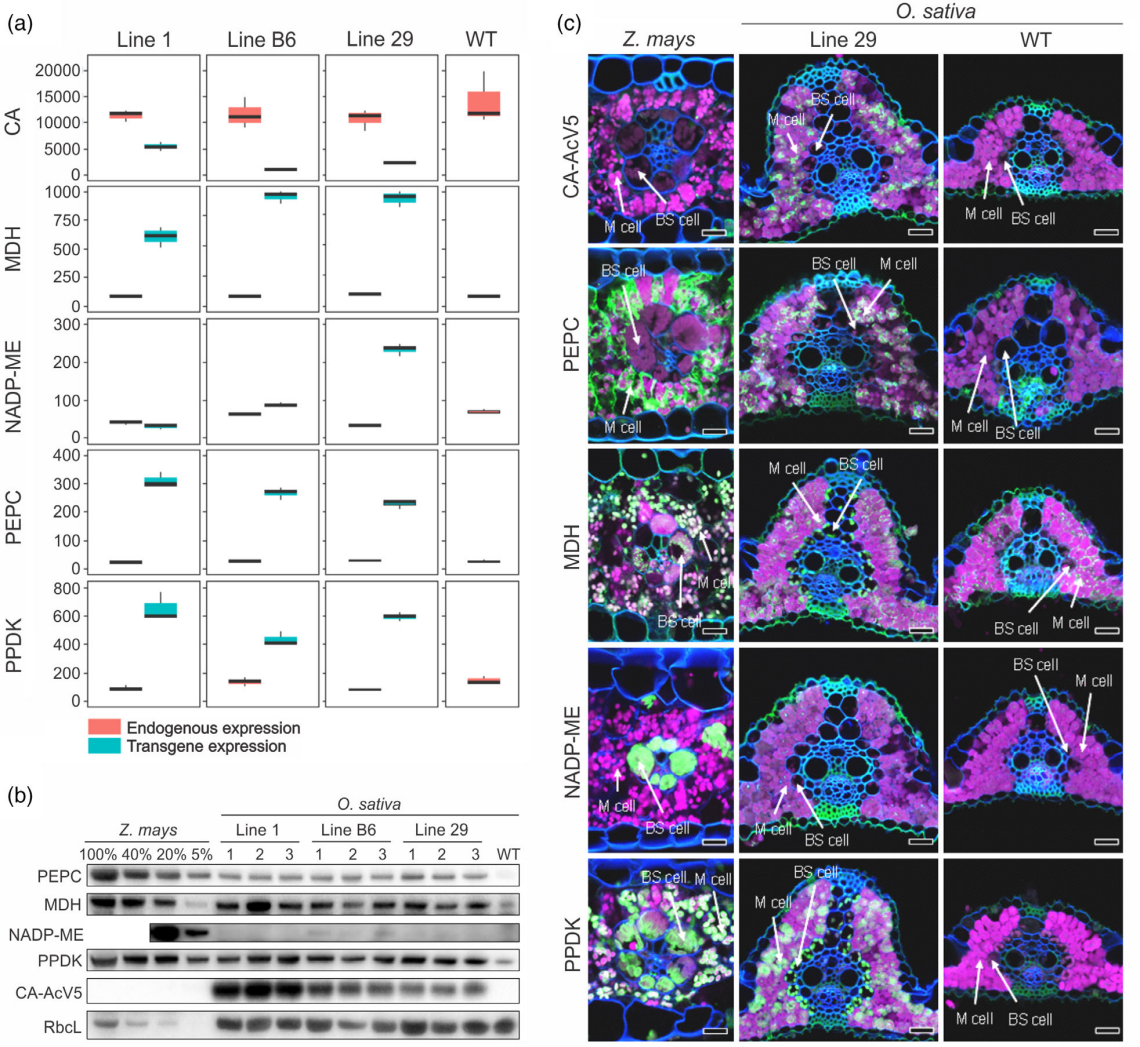
Expression of C_4_ enzymes in *O. sativa*. (a) Transcript abundance (in transcripts per million) of *Z. mays* transgenes and orthologous to them endogenous genes in wild‐type (WT) and three transgenic rice lines. CA, carbonic anhydrase; MDH, NADP‐malate dehydrogenase; NADP‐ME, NADP‐dependent malic enzyme; PEPC, PEP carboxylase; PPDK, pyruvate orthophosphate dikinase. Mean ± SD, *n* = 3 biological replicates. (b) Immunodetection of proteins in leaf extracts loaded on leaf area basis*. Z. mays* leaf extract dilution series was used for relative quantification; three plants from each transgenic line were analysed. Signal from RbcL (the large subunit of ribulose bisphosphate carboxylase oxygenase) was used as loading control. (c) Confocal micrographs of protein localization on leaf cross‐sections. Fluorescence signals are pseudo‐coloured: green ‐ protein of interest labelled with secondary antibodies conjugated with Alexa Fluor 488; magenta ‐ chlorophyll autofluorescence; blue ‐ calcofluor white‐stained cell walls. BS, bundle sheath; M, mesophyll. Scale bars = 20 µm. Localization of C_4_ enzymes in transgenic lines 1 and B6 is presented in Figure S1 and the summary of localization is presented in Table S1.

Transgene transcript abundance was analysed by RNA sequencing. Figure 1a reveals some variation in transgene expression between the lines with line 1 having the highest levels of *ZmCA* transcripts but about two‐fold lower levels of *ZmMDH* transcripts in comparison with lines 29 and B6. Across all three transgenic lines, *ZmCA* transcript abundance was highest, followed by *ZmMDH*, *ZmPPDK*, *ZmPEPC* and *ZmNADP‐ME*, with *ZmNADP‐ME* transcripts detectable above background only in lines 29 and B6. Transcript abundance of the endogenous rice orthologs of the *Z. mays* transgenes did not change in response to transgene expression in the three transgenic lines.

Accumulation of C_4_ enzymes was confirmed by immunoblotting of leaf protein extracts (Figure 1b). When compared to wild‐type (WT) plants, all transgenic lines accumulated higher levels of PEPC, MDH and PPDK. These abundances correspond to 20‐60% of levels in *Z. mays* (per leaf area) for MDH and 3‐5% for PEPC and PPDK. Consistent with the transcript abundance data, CA protein abundance was also highest in line 1. NADP‐ME protein was only just detectable in lines B6 and 29 and not detectable in line 1. In accordance with protein abundance, enzyme assays demonstrated significantly increased activities of PEPC, MDH and PPDK compared to WT in all three transgenic lines whereas NADP‐ME activity was only higher in lines B6 and 29 (Table 1). PEPC, MDH, NADP‐ME and PPDK activities were increased by up to three‐fold, 13‐fold, 15‐fold and seven‐fold compared to WT, reaching values that were up to 2%, 47%, 2% and 10% of those reported in maize, respectively. No increase in CA activity was detected in transgenic lines, likely due to the high‐activity levels of the endogenous chloroplast‐targeted CA isoform present in WT rice. Rubisco activity, chlorophyll content and leaf dry weight were all unaltered compared to WT (Table 1) and no growth phenotype was observed in the transgenic plants.

**Table 1:** Enzyme activity and leaf parameters determined on wild‐type (WT) *O. sativa* and three transgenic lines expressing enzymes of the C_4_ metabolic pathway. *Z. mays* enzyme activity rates are given for comparison

Parameter	*O. sativa*				*Z. mays*
	WT	Line 1	Line B6	Line 29	
Enzyme activity					
PEPC, µmol m^‐2^ s^‐1^	2.40 ± 0.23^a^	7.15 ± 0.32^b^	6.14 ± 0.66^b^	7.01 ± 1.02^b^	315.20
MDH, µmol m^‐2^ s^‐1^	11.18 ± 1.59^a^	104.01 ± 14.02^b^	147.93 ± 27.07^b^	152.87 ± 34.08^b^	323.93
NADP‐ME, µmol m^‐2^ s^‐1^	0.14 ± 0.03^a^	0.23 ± 0.05^a^	0.96 ± 0.05^c^	0.60 ± 0.03^b^	48.66
PPDK, µmol m^‐2^ s^‐1^	0.31 ± 0.10^a^	4.75 ± 0.33^c^	3.53 ± 0.27^b^	3.66 ± 0.14^b^	37.81
CA, mol m^‐2^ s^‐1^ bar^‐1^	15.74 ± 0.96^a^	15.01 ± 1.60^a^	15.67 ± 1.58^a^	16.22 ± 2.84^a^	9.71
Rubisco, µmol m^‐2^ s^‐1^	83.59 ± 5.63^a^	74.12 ± 7.40^a^	74.45 ± 6.13^a^	65.00 ± 11.45^a^	47.68
LMA, g (dry weight) m^‐2^	57.62 ± 0.22^a^	59.52 ± 0.15^a^	69.52 ± 0.55^a^	54.76 ± 0.33^a^	
Chlorophyll (*a* + *b*), mmol m^‐2^	0.53 ± 0.02^a^	0.53 ± 0.03^a^	0.52 ± 0.04^a^	0.55 ± 0.04^a^	
Chlorophyll *a*/*b*	4.57 ± 0.08^a^	4.44 ± 0.12^a^	4.48 ± 0.06^a^	4.65 ± 0.10^a^	

Mean ± SE, *n* = 4 biological replicates. Significance was evaluated by one‐way ANOVA and Tukey’s *post hoc* test, letters indicate significant differences between the groups (α> 0.05).

Abbreviations: CA, carbonic anhydrase; LMA, leaf mass per area; MDH, malate dehydrogenase; NADP‐ME, NADP‐dependent malic enzyme; PEPC, PEP carboxylase; PPDK, pyruvate orthophosphate dikinase; Rubisco, ribulose bisphosphate carboxylase oxygenase.

To determine whether the C_4_ enzymes accumulated in the correct cellular compartment, immunolocalization was performed with specific antibodies against the *Z. mays* enzymes or, in the case of CA, the AcV5 tag, using thin leaf cross‐sections and laser confocal microscopy (Figure 1c, Figure S1, Table S1). PEPC was correctly compartmentalized in the cytosol of M cells in lines 29 and B6 but was also present in BS cells in line 1; MDH was present in both cell‐types in all three lines rather than being M cell‐specific, as it would in a C_4_ plant; CA‐AcV5 was correctly localized to M cells in line 29 but was in both cell‐types in the other two lines; and NADP‐ME could not be detected in any of the lines. PPDK accumulated in both cell‐types in all three transgenic lines, as it does in maize (Majeran *et al*., 2005). Given these accumulation patterns, we predicted that carboxylation by PEPC would be possible in M cells of all three lines, as would conversion of oxaloacetate to malate by MDH. Metabolic consequences beyond those steps could not be predicted, but the inability to detect NADP‐ME in BS cells of the transgenic lines suggested that decarboxylation of malate was unlikely to occur.

### Detection of C_4_‐carboxylation

^13^CO_2_ labelling experiments were performed to assess whether there was any C_4_ pathway flux in transgenic lines. Leaves from WT and transgenic plants were supplied with ^13^CO_2_ under ambient growth conditions using a custom‐designed labelling chamber (Figure S2; see Method S2). Isotopomers of C_4_ pathway intermediates and Calvin–Benson cycle (CBC) intermediates were quantified by reverse‐phase liquid chromatography, coupled to tandem mass spectrometry (LC‐MS/MS), after both ^13^CO_2_‐pulse and ^13^CO_2_‐pulse/^12^CO_2_ chase labelling experiments.

**Figure 2:**
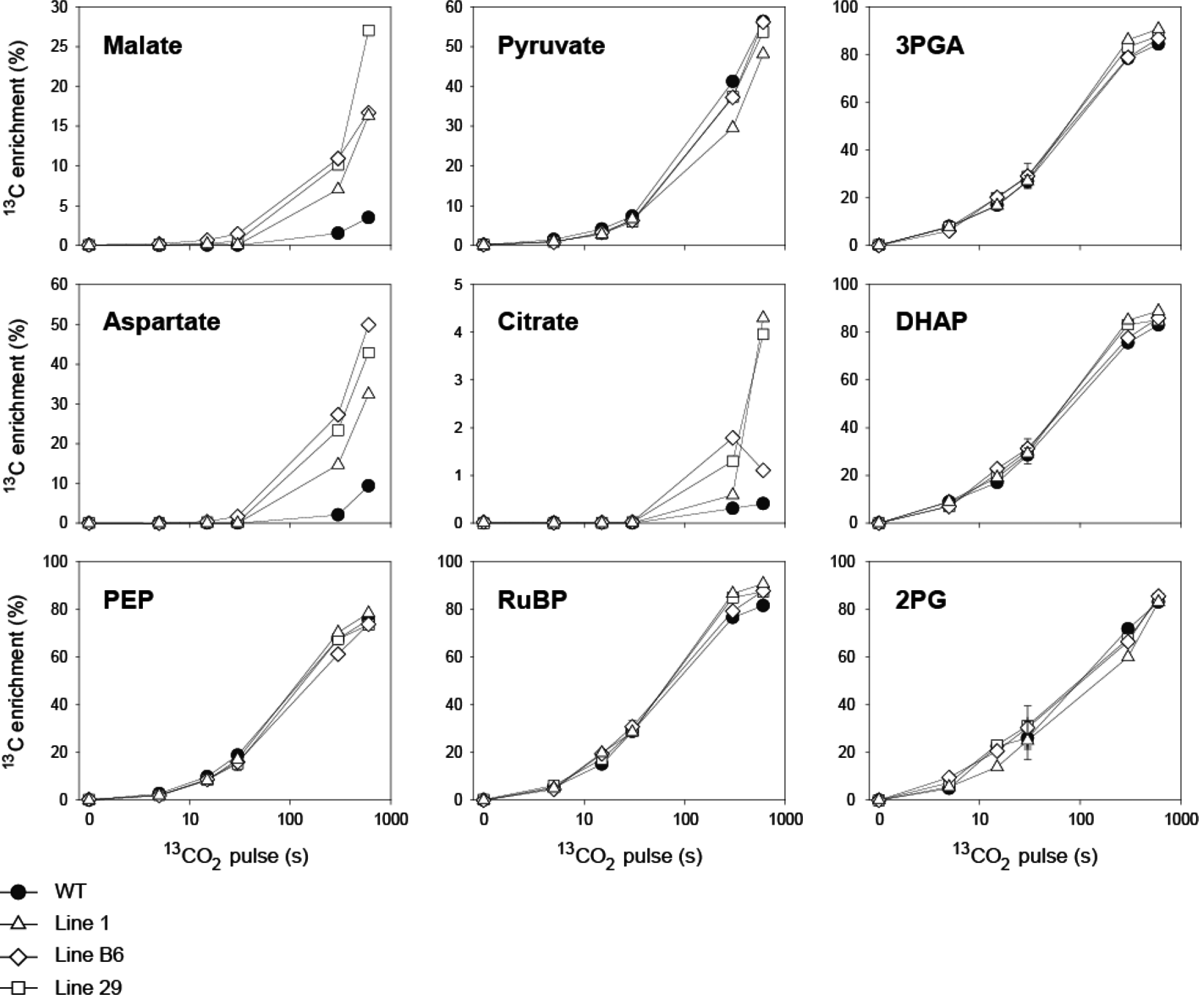
^13^C enrichment (%) during ^13^CO_2_‐pulse labelling of wild‐type (WT) rice and three transgenic lines expressing enzymes of the C_4_ metabolic pathway. 3PGA, 3‐phosphoglycerate; DHAP, dihydroxyacetone phosphate; PEP, phosphoenolpyruvate; RuBP, ribulose‐1,5‐bisphosphate; 2PG, 2‐phosphoglycolate. The *x*‐axes show the pulse labelling time on a log scale. Values from the 30 s time point are mean ± SD, *n* = 3‐4 biological replicates. Values at all other time points are from individual samples or means of two biological replicates. Abundances of individual isotopomers are shown in Figure 3 and Figure S9. The original data are presented in Data S1.

**Figure 3:**
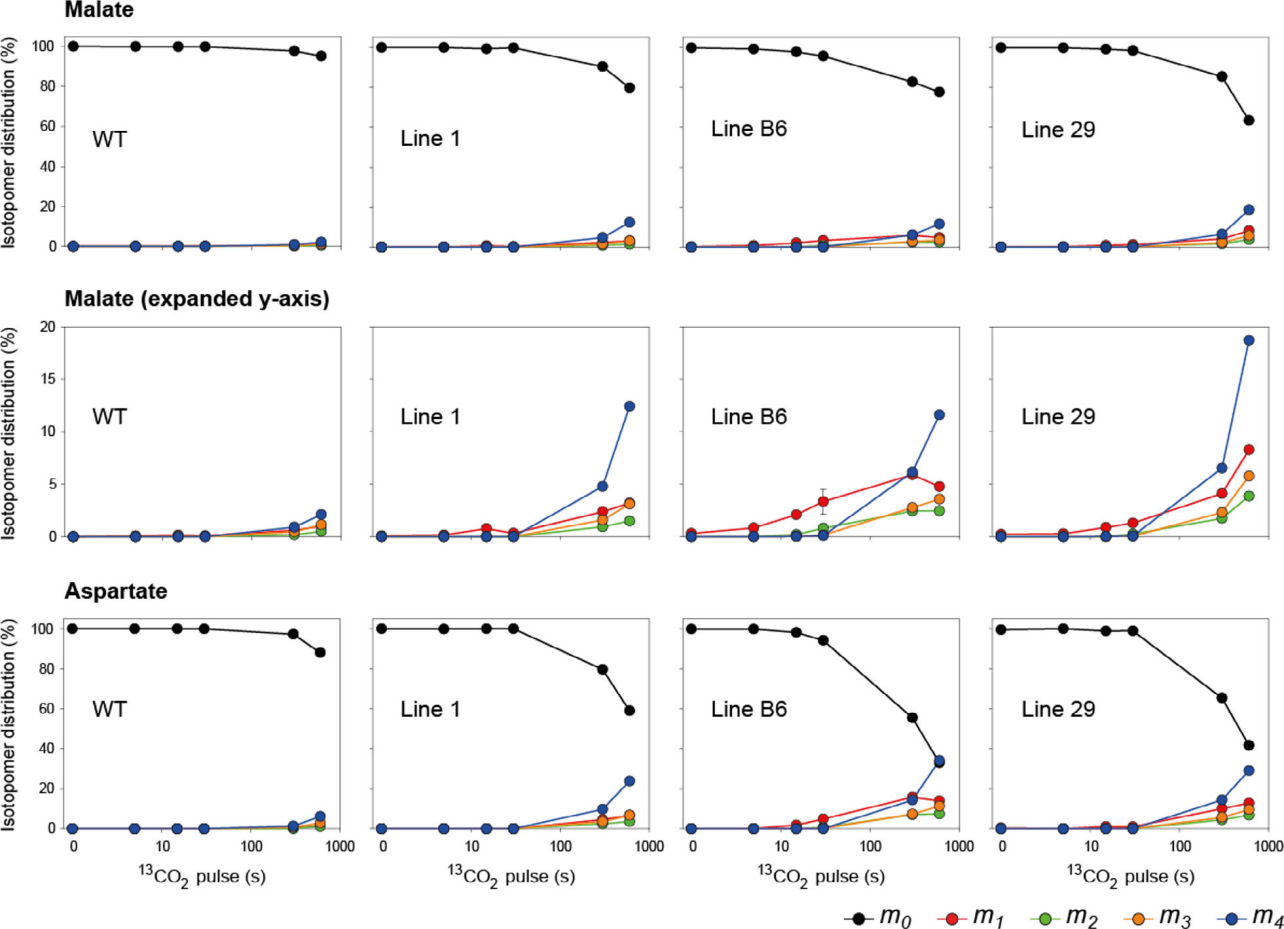
Isotopomer distribution (%) of malate and aspartate during ^13^CO_2_‐pulse labelling of wild‐type (WT) rice and three transgenic lines expressing enzymes of the C_4_ metabolic pathway. The relative abundance of each isotopomer (*m_n_*) for a given metabolite is represented, where *n* is the number of ^13^C atoms incorporated. The x‐axes show the pulse labelling time on a log scale. Values from the 30 s time point are mean ± SD, *n* = 3‐4 biological replicates. Values at all other time points are from individual samples or means of two biological replicates. Isotopomer abundances for further metabolites are shown in Figure S9. The original data are presented in Data S1.

When WT plants were pulse‐labelled with ^13^CO_2_, ^13^C enrichment rose rapidly in 3‐phosphoglycerate (3PGA) and other CBC intermediates ‐ dihydroxyacetone phosphate (DHAP) and ribulose‐1,5‐bisphosphate (RuBP) ‐ (up to 90% ^13^C enrichment after 600 s), and almost as rapidly in the photorespiratory intermediate 2‐phosphoglycolate (2PG), but there was little ^13^C enrichment in C_4_ acids, malate and aspartate (Figure 2; Figure S3). These labelling patterns are typical for C_3_ species, as shown previously in Arabidopsis (*Arabidopsis thaliana*), tobacco (*Nicotiana tabacum*) and cassava (*Manihot esculenta*) (Arrivault *et al*., 2019; Hasunuma *et al*., 2009; Ma *et al*., 2014; Szecowka *et al*., 2013). By contrast, ^13^C enrichment of malate rose faster in the three transgenic rice lines than in WT (Figure 2), with the *m_1_* isotopomer as well as the *m_2_* and *m_3_* isotopomers being more abundant than in WT (Figure 3). Line 29 had the highest ^13^C enrichment in malate (Figure 2, Figure 3). Similar results were observed for aspartate where line B6 had the highest enrichment (Figure 2, Figure 3). Calculation of ^13^C enrichment half times confirmed the faster rise in ^13^C enrichment of malate and aspartate in all three transgenic lines (Table S2).

Rates of ^13^C accumulation, estimated from the initial slope of net ^13^C accumulation plotted against ^13^C‐pulse labelling time, provided a proxy for minimum ^13^C fluxes (Figure S4; Data S1). ^13^C accumulation rates were similar in the three transgenic lines and approximatively 10‐fold higher than in WT for malate and three‐fold higher for aspartate (Table S3). The summed rates of ^13^C accumulation in malate and aspartate in the transgenic lines were equivalent to up to 330 nmol ^13^C equivalents g^‐1^ FW h^‐1^, which is about 1.7% of a typical photosynthesis rate in maize under similar conditions (~190 µmol CO_2_ g^‐1^ FW h^‐1^) (Arrivault *et al*., 2016). Moreover, total measured metabolite pool sizes indicated that all three transgenic lines had significantly more malate (up to 50%) and less aspartate than the WT (Figure S5; Table S4).

The kinetics of ^13^C enrichment in PEP and 3PGA differs between C_3_ and C_4_ plants (Arrivault *et al*., 2019; Arrivault *et al*., 2016) and the ratio of [^13^C enrichment in PEP]: [^13^C enrichment in 3PGA], termed hereafter the PEP:3PGA enrichment ratio, can provide evidence for the operation of C_4_ cycle (Figure S6). In WT rice the enrichment rose more slowly in PEP than 3PGA, similar to cassava (Arrivault *et al*., 2019). In maize, enrichment rose slower in PEP than 3PGA (data from (Arrivault *et al*., 2016) is replotted in Figure S6) reflecting a large flux of unlabelled C from C_4_ cycle intermediates into PEP (see legend of Figure S6). The kinetics of the PEP:3PGA enrichment ratio in the three transgenic rice lines broadly resembled that of WT rice, suggesting that PEP was mostly labelled from 3PGA via the reversible reactions catalysed by phosphoglyceromutase and enolase (Furbank and Leegood, 1984). However, the enrichment ratio was slightly lower at the first time points suggesting the operation of C_4_ cycle at a low level (Figure S6, significant for lines B6 and 29). Calculation of ^13^C enrichment half times confirmed the slightly slower rise in enrichment in PEP in lines B6 and 29 compared to WT rice (Table S2).

No consistent evidence of loss of label from malate or aspartate in the transgenic lines and no increase in ^13^C labelling of 3PGA or other CBC intermediates were found during the pulse/chase labelling (Figures S7 and S8). These results were corroborated by the pulse labelling kinetics and the rates of ^13^C accumulation in RuBP, DHAP, pyruvate and 2PG that were broadly similar between the transgenic and WT plants (Figure 2, Figure S9). The transgenic lines had more PEP and RuBP than the WT plants, but there were no significant differences in 3PGA, DHAP, pyruvate and 2PG content (Figure S5, Table S4). Movement of the ^13^C label to the citric acid cycle in mitochondria was evident from the ^13^C labelling of citrate being readily detectable in the three transgenic lines but not in WT rice (Figure 2, Figure S9). Calculation of half times confirmed the faster rise in ^13^C enrichment of citrate in all three transgenic lines (Table S2). This enrichment was low in percentage terms and no significant differences in total citrate amount were detected (Figure S5, Table S4).

### Effects of enhanced C_4_‐carboxylation on C_3_ photosynthesis

**Figure 4:**
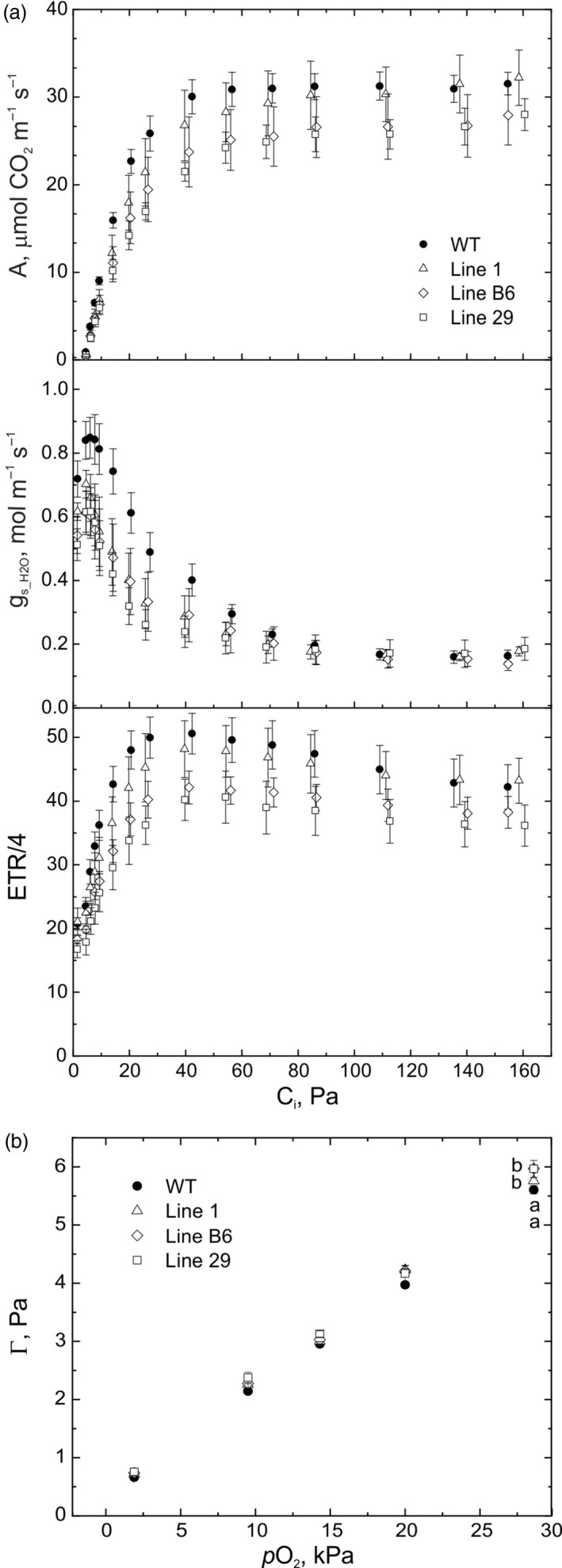
Gas‐exchange and fluorescence analysis of wild‐type (WT) *O. sativa* and three transgenic lines expressing enzymes of the C_4_ metabolic pathway. (a) A, net CO_2_ assimilation rate; g_s_H2O_, leaf stomatal conductance to water vapour; ETR/4, light‐driven electron transport rate through PHotosystem II divided to four, measured at different intercellular *p*CO_2_ (C_i_), PPFD of 1500 µmol m^‐2^ s^‐1^ and ambient *p*O_2_ (20 kPa). Parameters determined by fitting the A‐C_i_ response curves and statistical analysis are provided in Table 2. (b) Leaf CO_2_ compensation point (Г). Mean ± SE, *n* = 5 biological replicates for WT, *n* = 4 otherwise. Statistical analysis was performed using one‐way ANOVA and Tukey’s *post hoc* test, letters indicate significant differences between the groups (α > 0.05) .

To determine whether the altered metabolism observed in transgenic lines had a fitness cost, we carried out comprehensive gas‐exchange and fluorescence analysis. At steady‐state, when leaves were adapted to photosynthetic photon flux density (PPFD) of 1500 µmol m^‐2^ s^‐1^, no significant differences in net CO_2_ assimilation rate, stomatal conductance to H_2_O or the ratio between intercellular and ambient *p*CO_2_ were detected between the transgenic lines and WT (Table 2). The response of CO_2_ assimilation rate, stomatal conductance and Photosystem II (PSII) electron transport rate (ETR) to varying intercellular *p*CO_2_ for the three transgenic lines were similarly not significantly different from WT (Figure 4a), nor were the maximum carboxylation rate of Rubisco or the rate of photosynthetic electron transport (Table 2). The rate of respiration in the dark was not affected in the transgenic rice lines (Table 2).

**Table 2:** Gas‐exchange and fluorescence parameters of wild‐type (WT) *O. sativa* and three transgenic lines expressing enzymes of C_4_ metabolic pathway

Parameter	WT	Line 1	Line B6	Line 29
R_d_, µmol CO_2_ m^‐2^ s^‐1^	1.05 ± 0.05^a^	1.11 ± 0.22^a^	1.12 ± 0.10^a^	1.05 ± 0.04^a^
F_V_/F_M_	0.79 ± 0.01^b^	0.78 ± 0.01^b^	0.75 ± 0.01^a^	0.77 ± 0.01^ab^
C_i_/C_a_	0.74 ± 0.01^a^	0.75 ± 0.02^a^	0.75 ± 0.02^a^	0.76 ± 0.02^a^
A, µmol CO_2_ m^‐2^ s^‐1^	29.0 ± 1.2^a^	26.5 ± 2.7^a^	23.7 ± 2.3^a^	22.2 ± 1.9^a^
g_s_H2O_, mol H_2_O m^‐2^ s^‐1^	0.60 ± 0.05^a^	0.55 ± 0.05^a^	0.48 ± 0.05^a^	0.45 ± 0.03^a^
*V*_*cmax*_, µmol CO_2_ m^‐2^ s^‐1^	132 ± 12^a^	111 ± 20^a^	95 ± 18^a^	88 ± 13^a^
*J*, µmol e^‐^ m^‐2^ s^‐1^	144 ± 8^a^	140 ± 16^a^	121 ± 17^a^	119 ± 8^a^
*J*/*V*_*cmax*_	1.11 ± 0.06^a^	1.32 ± 0.11^a^	1.32 ± 0.12^a^	1.39 ± 0.12^a^
*TPU*, µmol CO_2_ m^‐2^ s^‐1^	10.6 ± 0.4^a^	11.0 ± 1.0^a^	9.4 ± 1.1^a^	9.5 ± 0.6^a^

Leaf dark respiration rates (R_d_) and the maximum quantum efficiency of Photosystem II (F_V_/F_M_) were determined after 40‐min dark adaptation. Ratio between intercellular and ambient *p*CO_2_ (C_i/_C_a_), net CO_2_ assimilation rates (A) and stomatal conductance to H_2_O (g_s_H2O_) were determined in steady‐state conditions at 1500 µmol m^‐2^ s^‐1^ PPFD and C_a_ = 37 Pa. Maximum carboxylation rate allowed by Rubisco (*V*_*cmax*_ *)*, rate of photosynthetic electron transport based on NADPH requirement (*J*), and triose phosphate use (*TPU*) were determined by fitting the A‐C_i_ response curves (Figure 4a). Mean ± SE, *n* = 5 biological replicates for WT, *n* = 4 otherwise. Statistical analysis was performed using one‐way ANOVA and Tukey’s *post hoc* test, letters indicate significant differences between the groups (α > 0.05).

Since engagement of the C_4_ cycle in transgenic rice was predicted to influence the CO_2_ compensation point (Ermakova *et al*., 2020), this parameter was determined at different atmospheric O_2_ levels (Figure 4b). At *p*O_2_ between 1.9 and 20 kPa, the CO_2_ compensation points of transgenic lines did not differ from WT. However, at *p*O_2_ of 28.6 kPa, above the ambient level, lines B6 and 29 had a significantly higher CO_2_ compensation point compared to WT rice and line 1 (Figure 4b). In addition, the maximal dark‐adapted quantum efficiency of PSII, which is often decreased in plants subjected to various stresses (Murchie and Lawson, 2013), was significantly lower in line B6 (Table 2). These results pointed to a potentially lower resilience of the transgenic plants to stress conditions.

**Figure 5:**
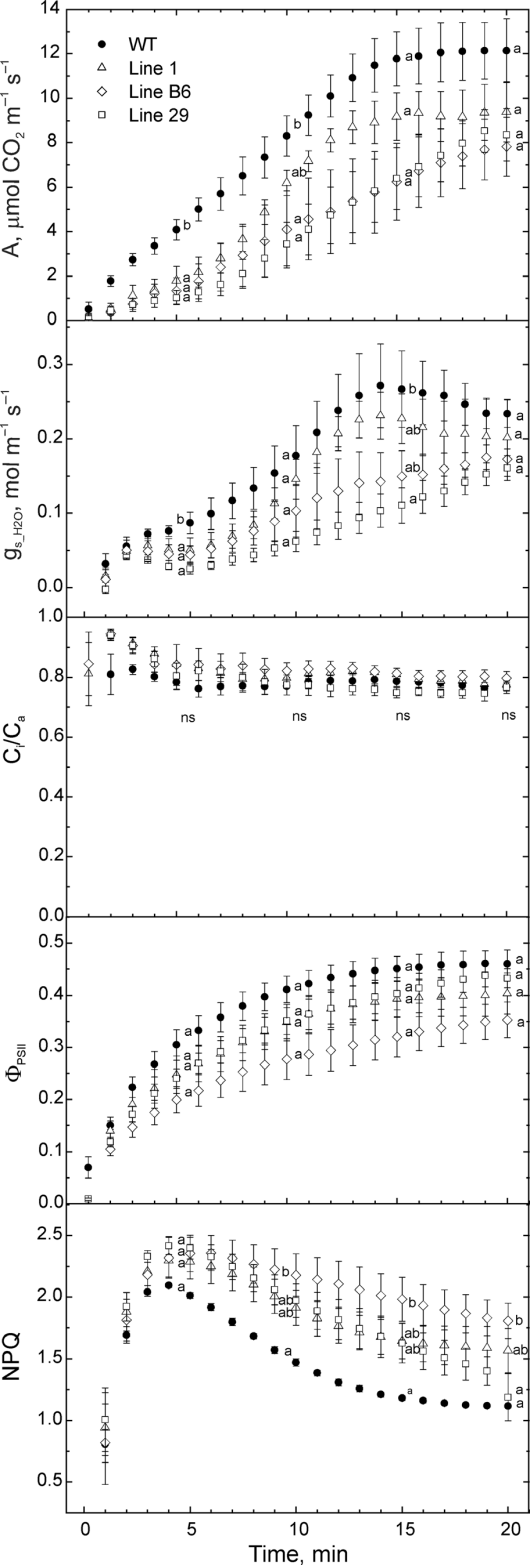
Gas‐exchange and fluorescence analysis of wild‐type (WT) *O. sativa* and three transgenic lines expressing enzymes of the C_4_ metabolic pathway grown at lower irradiance of about 200 µmol m^‐2^ s^‐1^ during the first 20 min after dark‐to‐light transition. Measurements were done on 40‐min dark‐adapted leaves at PPFD of 500 µmol m^‐2^ s^‐1^ and ambient *p*O_2_ (20 kPa). A, net CO_2_ assimilation rate; g_s_H2O_, leaf stomatal conductance to water vapour; C_i_/C_a_, ratio between intercellular and ambient *p*CO_2_; Φ_PSII_, quantum efficiency of Photosystem II; NPQ, non‐photochemical quenching. Mean ± SE, *n* = 5 biological replicates for WT, *n* = 4 otherwise (α> 0.05). Statistical analysis was performed at 5, 10, 15 and 20 min after the beginning of illumination using one‐way ANOVA and Tukey’s *post hoc* test, letters indicate significant differences between the groups (α > 0.05); ns, not significant.

To further explore these effects, we studied the induction of photosynthesis over the first 20 min of illumination on plants grown under normal (400 µmol photons m^−2^ s^−1^) and low irradiance (200 µmol photons m^−2^ s^−1^). No significant differences in CO_2_ assimilation rate, stomatal conductance, Φ_PSII_ (the effective quantum yield of PSII) and non‐photochemical quenching (NPQ, a measure of the absorbed light energy that is actively dissipated as heat in the PSII antennae) were found between the plants grown under normal light (Figure S10). However, when grown at a lower irradiance, all three transgenic lines had a slower induction of CO_2_ assimilation rate and stomatal conductance during the first 5 min of illumination (Figure 5). After 10 min of illumination, lines B6 and 29 still showed reduced CO_2_ assimilation rates whereas after 15 min neither of the transgenic lines differed from WT. Stomatal conductance in plants of line 29 was still lower than in WT after 15 min of illumination but after 20 min this difference was greatly diminished (Figure 5). Importantly, the ratio of intercellular and ambient *p*CO_2_ did not differ between the lines and WT indicating that stomatal conductance did not impose direct limitations to the assimilation rate. Although differences in Φ_PSII_ were not significant, NPQ was significantly higher in all three transgenic lines after 5 min of illumination and in line B6 NPQ remained significantly higher also after 20 min of induction (Figure 5).

### Differentially regulated genes

Given that overexpression of functional C_4_ enzymes, and the concomitant establishment of C_4_‐carboxylation, produced minimal effects on the physiological response of the C_3_ leaf we sought to determine whether the lack of phenotypic response was attributable to compensatory mechanisms arising through altered gene expression. In addition, we investigated whether alteration of the expression of genes encoding enzymes or transporters in related biochemical pathways might help to explain the lack of a phenotypic perturbation or provide insight into why a fully operational C_4_ cycle was not established. To address these questions, WT and transgenic plants were subject to transcriptome sequencing and differential expression analysis.

In total, only 58 genes (0.2% of all genes) were consistently differentially expressed when transgenic lines were compared to WT control (Figure S11). Thus, the transcriptome phenotype is minimally perturbed, mirroring the subtle physiological phenotype. Although several genes that are differentially expressed lack functional annotations, none of the detected genes with functional annotations encode proteins that are known to transport or convert metabolites of the core C_4_ cycle. Thus, compensatory changes to the transcriptome that could divert malate from being decarboxylated in BS chloroplast did not occur.

Although changes to the expression of genes encoding C_4_ cycle‐related enzymes and transporters were not detected, shared changes in gene expression of several photosynthesises‐related genes were observed (Table S5). These include genes whose products function in the regulation of stomatal movement, photomorphogenesis, the circadian clock, thioredoxin(‐like) genes and multiple genes involved in the production of xanthophyll. Collectively these may contribute to the moderate effect on CO_2_ assimilation that was observed. Moreover, it is noteworthy that the rice ortholog of the Arabidopsis gene *PROTEIN DISULFIDE ISOMERASE 6*, which is an attenuator of D1 protein synthesis facilitating photoinhibition in response to high light (Wittenberg *et al*., 2014), is upregulated in the transgenic lines. Enhanced susceptibility to photoinhibition may also help to explain the enhanced NPQ and slightly decreased maximum PSII efficiency observed in the transgenic lines.

## Discussion

### Multiple C_4_ enzymes can be functionally expressed in rice from a single construct

Engineering C_4_ photosynthesis into C_3_ plants is proposed to be a major way to increase radiation, nitrogen and water‐use efficiencies and consequently crop yield (Hibberd *et al*., 2008; Schuler *et al*., 2016). In this study, we used a single construct to introduce five enzymes of the C_4_ pathway into rice, exploiting previously reported cell‐preferential promoters to drive expression of maize cDNA sequences (Engelmann *et al*., 2008; Gupta *et al*., 2020). Less than half of the screened events showed expression of all five transgenes (Figure S12), possibly due to deletion of key regulatory elements by recombination events either in agrobacteria or during the T‐DNA integration. Protein levels (Figure 1b) and extractable enzyme activity (Table 1) in homozygous segregants from three independent homozygous lines reflected relative transcript levels (Figure 1a) and were consistent with previous reports of expression from cDNAs encoding C_4_ enzymes (Fukayama *et al*., 2001; Taniguchi *et al*., 2008). However, protein levels were substantively lower than those seen when genomic gene sequences were expressed in rice (Miyao *et al*., 2011) suggesting that intron sequences or other untranslated regions may elevate levels of transcripts, and hence protein, in the context of the rice leaf. In addition, considerable variation in gene and protein expression between the three transgenic lines demonstrated that genomic context and selective down‐regulation of some transgenes in the T‐DNA may affect expression patterns.

Of the five C_4_ enzymes, NADP‐ME showed the least consistent and lowest protein accumulation levels in all three transgenic lines. The NADP‐ME cDNA sequence was driven by the *GLDP* promoter shown to be active in bundle sheath and vascular cells (Engelmann *et al*., 2008). Although the strength of this promoter relative to the M‐preferential *PEPC* promoter is unknown, line B6 did have appreciable *NADP‐ME* expression at the transcript level, detectable NADP‐ME protein by immunoblotting, and an almost seven‐fold increase in enzyme activity above the albeit very low basal value in WT (Figure 1, Table 1). However, the level of increased activity was insufficient to detect decarboxylation of malate *in vivo*.

### Operation of a partial C_4_ cycle in rice

Labelling of C_4_ acids—malate and aspartate—was consistently higher in the three independent transgenic lines than in WT rice plants (Figure 2, Figure 3), indicating substantially increased fixation of CO_2_ by PEPC in lines expressing the *Z. mays* PEPC enzyme. Rates of ^13^C accumulation in malate were similar in the three transgenic lines and approximatively 10‐fold higher than in WT (Table S3). This increase in estimated flux was larger than the 2.5‐ to three‐fold increase in PEPC activity and resembled the 10‐fold increase in MDH expression in these lines (Table 1). This over‐proportional increase in flux indicates that *Z. mays* PEPC can operate efficiently *in vivo* in rice, perhaps more efficiently than the endogenous rice PEPC due to the altered kinetic properties of the C_4_ enzyme, that is, insensitivity to inhibition by post‐translational modification and product‐inhibition by malate (Endo *et al*., 2008). Nonetheless, the estimated flux at PEPC in the transgenic rice lines was only about 2% of that required during photosynthesis in maize and thus corresponding to the *in vitro* PEPC activity (Table 1). Thus, a considerably larger increase in PEPC expression will likely be required to establish C_4_ photosynthesis in rice.

The pulse‐chase experiments provided no compelling evidence for C_4_ acid decarboxylation by the introduced *Z. mays* NADP‐ME or for re‐fixation of CO_2_ into CBC intermediates in the transgenic lines. It should be noted that the ^13^CO_2_ pulse was kept short (30 s) so that the *m_1_* isotopomers of malate and aspartate were the most abundant labelled isotopomers (Hatch, 1971). With such a short pulse, ^13^C enrichments for malate and aspartate were less than 2% in the transgenic lines and likely too low to detect their decarboxylation. Another confounding factor is that there was already substantial labelling of the CBC intermediates by the end of the 30 s pulse (around 30%), so movement of ^13^C from the weakly labelled pools of C_4_ acids into CBC intermediates would have been difficult to detect above this background. However, a slight delay in labelling of PEP in the transgenic lines, potentially caused by a flux of unlabelled C from pyruvate into PEP at early times during a pulse (Figure S6, see also Arrivault *et al*., 2016; Hatch, 1971; Hatch and Slack, 1966), indicated that a C_4_ cycle may be operating, albeit at very low rates. This label disequilibrium between 3PGA and PEP may provide a proxy for flux around the C_4_ cycle, as suggested by the significant changes in lines B6 and 29, which had the largest increase in NADP‐ME activity. Consistently higher ^13^C labelling of citrate in all three transgenic lines (Figure 2, Figure S9) could be explained by movement of ^13^C‐labelled C_4_ acids (malate, oxaloacetate or fumarate) from the cytosol into the mitochondria and the citric acid cycle. Although small in absolute terms, entry of organic acids into the citric acid cycle could lead to futile cycling of C_4_ acids and have an adverse impact on operation of a photosynthetic C_4_ cycle.

### C_4_‐carboxylation has minimal effects on C_3_ leaf fitness

Previous studies examining physiological phenotypes in rice overexpressing individual enzymes of the C_4_ cycle have struggled to find effects (Giuliani *et al*., 2019; Miyao *et al*., 2011; Taniguchi *et al*., 2008), except for rice expressing the *Z. mays* NADP‐ME cDNA driven by the Arabidopsis chlorophyll *a*/*b*‐binding protein promoter (Takeuchi *et al*., 2000; Tsuchida *et al*., 2001), which severely inhibited photosynthesis and growth. Indeed, this has led authors to propose that high levels of activity and expression of C_4_ cycle enzymes in a C_3_ leaf is neutral under normal growth conditions (Karki *et al*., 2020). Fukayama *et al*. (2003) proposed that in rice with high‐level expression of maize PEPC, flux through PEPC *in vivo* was low, as PEP levels were limiting. Similarly, MDH overexpression alone had little physiological effect, potentially because of low availability of oxaloacetate in chloroplasts (Kandoi *et al*., 2018). Conceivably, concurrent overexpression of five C_4_ enzymes was required to detect ^13^C flux into malate, as well as the photosynthetic phenotype seen here. Synthesis of malate in the transgenic rice lines produced here presumably occurred in chloroplasts and consumed NADPH produced by electron transport. This additional consumption of NADPH could potentially inhibit CO_2_ fixation and deplete reducing power required for other reactions. All three transgenic lines showed an extended photosynthetic dark‐to‐light induction (Figure 5), and the severity of this phenotype was greater in lines B6 and 29 showing higher CO_2_ flux to C_4_ acids (Figure 3). This extended induction phase could be related to slower opening of stomata (Lawson and Blatt, 2014), autocatalytic build‐up of metabolites (Walker, 1973) or delayed light‐activation of enzymes (Anderson *et al*., 1978). Although enzymes of the C_4_ cycle establish guard cell turgor (Santelia and Lawson, 2016), stomatal conductance is not the primary cause of this phenomenon since the C_i_/C_a_ ratio was similar in all plants (Figure 5). Depletion of reducing power, however, could down‐regulate activity of the chloroplast thioredoxin reductase that uses NADPH to activate CBC enzymes and ATP synthase specifically during the dark‐to‐light transition and under low light (Nikkanen *et al*., 2018; Yoshida and Hisabori, 2016). This reduced activation could contribute to the build‐up of ΔpH and result in the slower NPQ relaxation observed in the transgenic lines (Figure 5). Additionally, reduced F_V_/F_M_ and the strongest NPQ phenotype during the induction seen in line B6 (Table 2, Figure 5) could be a result of the enhanced NADP‐ME abundance seen in this line, leading to a mild version of the chlorotic phenotype reported by Tsuchida *et al*. (2001).

in C_3_ plants, chloroplastic MDH is part of the malate shuttle; it contributes to photoprotection by oxidizing stromal electron acceptors (Scheibe and Stitt, 1988) and enables regulation of gene expression by equilibrating the redox state between cellular compartments (Dietz *et al*., 2016). C_3_ MDH is regulated by thioredoxin *m* and thus is active only when NAPDH accumulates and the thioredoxin system is pushed into a more reduced state (Collin *et al*., 2003; Scheibe, 1987). In C_4_ plants, the activation state of MDH is dynamically regulated by the ratio of NADPH to NADP^+^ (Ashton and Hatch, 1983; Rebeille and Hatch, 1986). The higher malate efflux from chloroplasts may potentially contribute to the differential regulation of multiple genes involved in light sensing, stomatal regulation and the circadian clock observed in the RNA profiles of the transgenic lines (Table S5). In summary, taken together these results indicate that the C_4_ photosynthetic enzymes expressed in the transgenic lines are functional *in vivo* and catalyse a partial C_4_ pathway.

### Concluding comments

For the first time, we demonstrate that a partial C_4_ pathway can be established in rice by transformation with a single construct harbouring coding sequences for five enzymes of C_4_ metabolism. Whilst expression levels of these enzymes require improvement, the cell‐specific expression patterns were largely appropriate for two‐cell C_4_ photosynthesis and the observed photosynthetic phenotypes of the transgenic plants were consistent with C_4_‐carboxylation occurring *in vivo*. These results suggest that a full C_4_ metabolic pathway may be achievable in rice.

## Materials and methods

### Generation of transgenic plants

Two constructs, EC18089 and EC18089B (Figure S12), were assembled in a plant binary vector pAGM4723 using the Golden Gate MoClo Plant Parts Kit (Engler *et al*., 2014). The first expression module was occupied either by the hygromycin phosphotransferase gene (*hpt*) in EC18089 or bialaphos resistance gene (*bar*) in EC18089B, in both cases the *Z. mays* ubiquitin promoter. Other modules were identical in the two constructs. The second module was occupied by the coding sequence of *ZmPEPC* (GRMZM2G083841) driven by the *Panicum miliaceum PEPC* promoter + 5’‐UTR (Gupta *et al*., 2020). The third module was occupied by the coding sequence of *ZmMDH* (GRMZM2G129513) under the control of the *Z. mays PEPC* promoter (Matsuoka *et al*., 1994). The fourth module was occupied by the coding sequence of *ZmNADP‐ME* (GRMZM2G085019) under the control of the *F. trinervia GLDP* promoter (Engelmann *et al*., 2008). The fifth module was occupied by the coding sequence of *ZmPPDK* (GRMZM2G097457) driven by the *Setaria viridis PEPC* promoter + 5’‐UTR (Gupta *et al*., 2020). The sixth module was occupied by the coding sequence of *ZmCA* (GRMZM2G348512) driven by the *Urochloa maxima PEPC* promoter + 5’‐UTR (Gupta *et al*., 2020). All coding and promoter sequences were domesticated for the Golden Gate cloning system (Engler *et al*., 2014). The bacterial terminator tNos was used in all modules. Both constructs were verified by sequencing and transformed into *Agrobacterium tumefaciens* strain AGL1 for stable rice transformation (Toki *et al*., 2006) as described in detail in Method S1.

T_0_ plants were analysed for insertion copy number of the *hpt* or *bar* genes by droplet digital PCR (iDNA genetics, UK). T_0_ plant of line 1 carried three copies of *hpt*, whilst T_0_ plants of lines 29 and B6 had one copy of *hpt* and *bar*, respectively. Analysis of *hpt* copy number in the T_1_ progeny of line 1 suggested that the three insertions segregated as a single genetic locus. Plants containing homozygous insertions were identified in T_1_ progenies of the three transgenic lines and seeds of those plants were thereafter used in all experiments. From a total of 47 independent transformation events, 25 were examined, and of these, less than half showed expression of all five genes at the transcript level (Figure S13).

### Plant growth conditions

Rice plants were grown in a controlled environment chamber (Model PGC Flex, Conviron, Winnipeg, MB, Canada) under ambient CO_2_ partial pressure, 16‐h photoperiod, 28 °C day, 22 °C night and 60% humidity. Irradiance of 400 μmol photons m^−2^ s^−1^ (if not stated otherwise) was supplied by a mixture of fluorescent tubes (Master TL5 HO 54W/840, Philips Lighting, The Netherlands) and halogen incandescent globes (42W 2800K warm white clear glass 630 lumens, CLA, Brookvale, Australia). Plants were individually grown in 1‐L pots in a soil mix composed by 80% peat/10% perlite/10% vermiculite (pH 5.6‐5.8) mixed with 5 g of slow‐release fertilizer (Osmocote, Evergreen Garden Care, Australia) supplied once at the beginning of the growth cycle. All pots were kept at field water capacity. *Z. mays* cv. B73 plants were grown in a controlled environment chamber using the same settings except that the light was supplied by 1000W red sunrise 3200K lamps (Sunmaster Growlamps, Solon, OH).

For ^13^CO_2_ labelling experiments, rice plants were grown in 1‐L pots in a 2:1 mixture of peat substrate and medium‐sized grain quartz sand (Einheitserdewerke Werkverband e.V, Sinntal‐Altengronau, Germany), containing 0.66 mL L^‐1^ Plantacote Depote 4M (Wilhelm Haug GmbH & Co. KG, Ammerbuch, Germany) and 0.66 mL L^‐1^ Fetrilon Combi (COMPO EXPERT GmbH, Muenster, Germany) fertilizer. Plants were grown in a controlled environment chamber with a 16‐h photoperiod and an irradiance of 350 μmol photons m^−2^ s^−1^ provided by LED lights, day/night temperatures of 26 °C/22 °C and constant 70% humidity. Pots were submerged in water. The plants were vegetatively propagated by detaching and re‐planting tillers at 35 days after sowing (DAS) and used for ^13^CO_2_ labelling experiments at 60 and 61 DAS. An additional ^13^CO_2_ labelling experiment was performed at 79 DAS.

### RT‐PCR and RNA sequencing

Leaf discs were collected from the mid‐distal leaf blade portion of the youngest fully expanded leaf from the central stem of 4‐week‐old rice plants, frozen in liquid N_2_ and stored at −80 °C. Frozen samples were homogenized using a Qiagen TissueLyser II (Qiagen, Venlo, The Netherlands). RNA was extracted using an RNeasy Plant Mini Kit (Qiagen, Venlo, The Netherlands). DNA from the samples was removed using an Ambion TURBO DNA free kit (Thermo Fisher Scientific, Tewksbury, MA) and RNA quality was determined using a NanoDrop (Thermo Fisher Scientific, Tewksbury, MA). For RT‐PCR, 200 ng of total RNA were reverse transcribed into cDNA using an RT^2^ HT First Strand cDNA synthesis kit (Qiagen, Venlo, The Netherlands). Primers listed in Table S6 were used for RT‐PCR and amplicons were visualized in 1% agarose gels.

For RNA sequencing, leaf discs from three plants were pooled together as one biological replicate and three biological replicates per genotype were analysed. cDNA synthesis, library preparation and sequencing were performed by BGI (https://www.bgi.com/). The raw sequence reads are available from EBI array express under the accession number E‐MTAB‐9129. Trimmomatic v0.39 (Bolger *et al*., 2014) was used to trim off the sequencing adapters and remove low quality bases using the following settings: LEADING:20 TRAILING:20 SLIDINGWINDOW:5:20 HEADCROP:1 MINLEN:35. The most recent version of the complete set of *O. sativa* transcripts (Osativa_323_v7.0.transcript.fa) was obtained from Phytozome V13 (Goodstein *et al*., 2011). The transcript sequences with correct 5’UTR and 3’UTR sequences (as per promoters and terminators used in the construct) corresponding to *ZmCA*, *ZmPEPC*, *ZmMDH*, *ZmPPDK* and *ZmME* were added to this file so that the transgenes could be simultaneously quantified with the rice transcriptome. The quality filtered trimmed reads were mapped to this modified reference transcriptome using Salmon (Patro *et al*., 2017). Prior to differential expression testing, the read counts for multiple isoforms of the same gene were summed to produce a single transcript abundance estimate per gene locus. These counts were then used as input to test for differential expression using DESeq2 v3.9 (Love *et al*., 2014). Genes were considered differentially expressed between WT and transgenic plants if the Benjamini–Hochberg adjusted *P*‐value was ≤0.01. The read counts for genes of the WT and transgenic rice lines are provided in Data S2. Accession numbers for endogenous rice genes that are orthologous to C_4_ transgenes and were used for comparison in Figure 1a are: OsKitaake01g256600.1.p (*CA*), OsKitaake08g251400.2.p (*MDH*), OsKitaake01g064200.1.p (*NADP‐ME*), OsKitaake02g105200.1.p (*PEPC*), OsKitaake05g157000.1.p (*PPDK*).

### Enzyme assays and immunodetection

Enzyme activities were determined using leaf extracts from fresh or frozen leaf tissue. For PPDK activity, a leaf disc was taken from the portion of leaf illuminated with the LI‐6800 (LI**‐**COR Biosciences, Lincoln, NE) during gas‐exchange measurements and immediately ground in a glass homogenizer in extraction buffer (Ashton *et al*., 1990; Voznesenskaya *et al*., 2003). PEPC activity was determined from the same fresh leaf extract (Pengelly *et al*., 2010). Additional leaf discs were frozen in liquid N_2_ and stored at −80 °C to later determine activities of Rubisco (Pengelly *et al*., 2010), NADP‐ME (Pengelly *et al*., 2012), MDH (Johnson and Hatch, 1970; Tsuchida *et al*., 2001) and CA (von Caemmerer *et al*., 2004). All enzyme activities were assayed at 25 °C and expressed per unit leaf surface area. Leaf chlorophyll content was determined spectrophotometrically in 80% acetone buffered with 25 mm 4‐(2‐hydroxyethyl)‐1‐piperazineethanesulfonic acid (Hepes)‐KOH (Porra *et al*., 1989).

For western blotting, aliquots of the leaf protein extracts used for enzyme activity measurements were supplemented with 2% (w/v) sodium dodecyl sulphate, separated by polyacrylamide gel electrophoresis and transferred to a nitrocellulose membrane according to (Ermakova *et al*., 2019). Proteins were probed with antisera against *Z. mays* PEPC (1:10,000 dilution, Karki *et al*., 2020), *Z. mays* PPDK (1:20,000 dilution, Karki *et al*., 2020), *Z. mays* MDH (1:5000 dilution, Karki *et al*., 2020), *Z. mays* NADP‐ME (1:5000 dilution, Karki *et al*., 2020; Sonawane *et al*., 2018), AcV5 tag (ab49581, Abcam, Cambridge, UK, 1:3000 dilution) and tobacco Rubisco (prepared by S.M. Whitney, 1:10,000). Secondary goat anti‐rabbit horseradish peroxidase‐conjugated (Biorad, Hercules, CA) or goat anti‐mouse horseradish peroxidase‐conjugated (Agrisera, Vännäs, Sweden) antibodies were used at a 1:5000 dilution. Chemiluminescence signal from the membranes was obtained with a Western Lightning Ultra kit (Perkin Elmer, Waltham, MA) and detected by a ChemiDoc MP imaging system (Biorad, Hercules, CA). Results were analysed in Image Lab software (Biorad, Hercules, CA). Details of immunolocalization of C_4_ enzymes performed on leaf sections are in Method S3. Proteins were visualized under a Leica SP8 laser scanning confocal microscope (Leica Microsystems GmbH, Wetzlar, Germany) at 488 nm excitation and 546 to 600 nm emission. Simultaneously, fluorescence from calcofluor white‐stained cell walls was detected at 434 to 445 nm following excitation at 405 nm. Chlorophyll autofluorescence was captured using 633 nm excitation and 650 to 742 nm emission. Images were analysed using LAS X software (Leica Microsystems GmbH, Wetzlar, Germany).

### Leaf gas‐exchange analysis

Leaf‐atmosphere CO_2_ and H_2_O exchange and chlorophyll fluorescence measurements were conducted with two portable photosynthesis systems LI‐6800 equipped with a Multiphase flash^TM^ fluorometer circular chamber (6800‐01A) as described in detail Method S4. The LI‐COR leaf chamber was set at PPFD of 1500 µmol photons m^−2^ s^−1^ (90% red/10% blue), leaf temperature of 25 °C, leaf**‐**to**‐**air vapour‐pressure deficit of 1.0 kPa and the airflow rate of 500 µmol s^−1^. Multiphase flash of 10,000 µmol photons m^‐2^ s^‐1^ (ramp 25%) was applied to leaves to transiently close all PSII reaction centres and monitor the maximum fluorescence in the light (F'_M_) and the steady‐state fluorescence (F_S_). Photochemical yield of PSII was determined as Φ_PSII_ = (F'_M_‐F_S_)/F'_M_ (Genty *et al*., 1989). The light‐driven electron transport rate through PSII was determined as ETR = α*PPFD*β*Φ_PSII_, where α is the leaf absorbance of photosynthetic quanta (0.843 according to Björkman and Demmig, 1987) and *β* is the fraction of photons absorbed by PSII (0.5 according to Maxwell and Johnson, 2000).

For dark‐to‐light induction measurements, leaves were dark‐adapted for 40 min and then enclosed in the dark fluorometer chamber for 10 minutes to measure dark respiration rate, the minimum PSII fluorescence (F_0_) and the maximum PSII fluorescence (F_M_). The maximum quantum yield of PSII was calculated as F_V_/F_M_ = (F_M_‐F_0_)/F_M_. Afterwards, the leaf was illuminated with actinic light of 500 µmol photons m^‐2^ s^−1^ to monitor photosynthetic induction. Non‐photochemical quenching was estimated as NPQ = (F_M_‐F'_M_)/F'_M_.

### Fitting of the A‐C_i_ response curves

The maximum leaf carboxylation rate allowed by Rubisco, the rate of photosynthetic electron transport and triose phosphate use were obtained by fitting the A‐C_i_ response curves (C_a_ steps from 0 to 190 Pa) at atmospheric *p*O_2_ using the fitting routine (Sharkey *et al*., 2007). Leaf mesophyll conductance to CO_2_ diffusion (g_m_) of 6.7 µmol CO_2_ m^‐2^ s^‐1^ Pa^‐1^ was previously determined for rice (von Caemmerer and Evans, 2015). For A‐C_i_ response curves recorded at different O_2_ levels, the least square regression method was applied to the initial slope (for C_i_ ≤ 9 Pa) to calculate the CO_2_ compensation point (Γ, Pa).

### ^13^CO_2_ labelling and quenching procedure

Labelling chambers were custom‐designed as shown in Figure S2. Details of chambers and the labelling procedure are described in detail in Method S2. Two sets of experiments were conducted: (i) ^13^CO_2_‐pulse labelling (0, 5, 15, 30, 300 and 600 s) was performed to determine the extent of ^13^C incorporation into C_4_ acids and other intermediates, and (ii) pulse‐chase labelling (30 s ^13^CO_2_‐pulse/0, 5, 15, 30, 60, 90 and 120 s ^12^CO_2_ chase) to detect movement of label out of C_4_ acids and into CBC intermediates. To gain a more complete overview of the temporal kinetics of ^13^C incorporation into each metabolite, we analysed single samples at multiple time points. Unlabelled samples (0) were also collected from leaves placed in the labelling chamber and flushed with the unlabelled air mixture for 1 min. The order of the ^13^CO_2_‐pulse labelling and pulse‐chase labelling times was randomized.

### Metabolite analyses and calculation of total pool size, ^13^C enrichment and isotopomer distributions

Frozen samples were homogenized using a ball mill (Tesch, Haan, Germany) at liquid nitrogen temperature. Chemicals used for quantification were from Sigma‐Aldrich (St. Louis, Missouri, USA), Roche (Basel, Switzerland) or Merck (Darmstadt, Germany). For LC‐MS/MS analysis, samples were extracted with chloroform–methanol as described in Arrivault *et al*. (2016). Isotopomers were measured by reverse‐phase LC‐MS/MS (malate, aspartate, PEP, RuBP, 3PGA, DHAP and 2PG; Arrivault *et al*., 2016) or anion‐exchange LC‐MS/MS (with modifications as described in Figueroa *et al*., 2016; pyruvate and citrate; Lunn *et al*., 2006) with authentic standards for accurate metabolite quantification. Total amounts of malate, aspartate, citrate, RuBP, DHAP and 2PG were calculated by summing isotopomers. The total amounts of PEP, pyruvate and 3PGA were determined enzymatically in freshly prepared trichloroacetic acid extracts as described in (Merlo *et al*., 1993), except for a modified assay buffer containing 50 mm Hepes‐KOH, pH 7.5, 200 mm KCl, 40 mm MgCl_2_ used for the determination of PEP and pyruvate. Net accumulation of ^13^C in each metabolite was calculated by multiplying the ^13^C enrichment by the number of C atoms in the molecule (*n*) and by the total amount of the metabolite. Net accumulation was plotted against ^13^C‐pulse labelling time (Figure S4) and the initial slope provided a proxy for minimum ^13^C fluxes (see Data S1 for calculation steps); these are a minimum estimate because some ^13^C may exit the metabolite pool during the pulse. Estimation of flux at PEPC is described in Data S1.

### Statistics and reproducibility

One‐way ANOVA and Tukey *post hoc* test for the pairwise comparisons of means (α = 0.05) was applied to all leaf traits determined in the present study using OriginPro 2018b software (OriginLab Corp., Northampton, MA). At least three biological replicates were used for each genotype for each measurement. At least ten sets of plants were grown for various experiments. Gas‐exchange, immunodetection of C_4_ enzymes, ^13^CO_2_ labelling and enzyme activity assays were partially replicated on different sets of plants.

## Conflict of interest

Authors declare no competing interests.

## Authors contribution

ABC, JAL, JEL, MS, SvC and RTF designed research. ME, SA, RG, FD, HAC, DV, HI, RF, MG, GLB performed research. ME, SA, RG, SK, JEL, MS, SvC and RTF analysed data. ML contributed research tools. ME, SA, RG, JAL, SK, JEL, MS, SvC and RTF wrote the paper.

## Supporting information


**Figure S1.** Confocal micrographs of C_4_ enzymes localization on leaf cross‐sections of *Z. mays* and *O. sativa* wild‐type (WT) and three transgenic *O. sativa* lines expressing C_4_ metabolic enzymes
**Figure S2.** ^13^CO_2_ labelling apparatus and quenching procedure
**Figure S3.** ^13^C enrichment (%) of wild‐type (WT) rice during (A) ^13^CO_2_‐pulse labelling and (B) pulse‐chase labelling
**Figure S4.** Net accumulation of ^13^C (nmol ^13^C equivalents g^‐1^ FW) during ^13^CO_2_‐pulse labelling of wild‐type (WT) rice and three transgenic lines expressing enzymes of the C_4_ metabolic pathway
**Figure S5.** Metabolite amounts (nmol g^‐1^ FW) of wild‐type (WT) rice and three transgenic lines expressing enzymes of the C_4_ metabolic pathway
**Figure S6.** Relative labelling of PEP and 3PGA during ^13^CO_2_‐pulse labelling of wild‐type (WT) rice, three transgenic lines expressing enzymes of the C_4_ metabolic pathway, maize and cassava
**Figure S7.** Pulse‐chase labelling of wild‐type (WT) rice and three transgenic lines expressing enzymes of the C_4_ metabolic pathway
**Figure S8.** Isotopomer distribution (%) during pulse‐chase labelling of wild‐type (WT) rice and three transgenic lines expressing enzymes of the C_4_ metabolic pathway
**Figure S9.** Isotopomer distribution (%) during ^13^CO_2_‐pulse labelling of wild‐type (WT) rice and three transgenic lines expressing enzymes of the C_4_ metabolic pathway
**Figure S10.** Gas‐exchange and fluorescence analysis of wild‐type (WT) *O. sativa* and three transgenic lines expressing enzymes of the C_4_ metabolic pathway during a dark‐to‐light shift
**Figure S11.** Number of differentially regulated genes in the three transgenic *O. sativa* lines expressing the C_4_ metabolic pathway compared to WT
**Figure S12.** Constructs used for stable rice transformation
**Figure S13.** RT‐PCR detection of *Z. mays* gene transcripts in T_0_ *O. sativa* lines transformed with the gene construct for C_4_ metabolic pathway expression
**Table S1.** Summary of C_4_ enzymes localization from the confocal images on Figure 1c and Figure S1.
**Table S2.** Estimation of ^13^C enrichment half times in different metabolites in wild‐type (WT) rice and three transgenic lines expressing enzymes of the C_4_ metabolic pathway during ^13^CO_2_‐pulse labelling.
**Table S3.** Estimation of minimum ^13^C fluxes using slopes of ^13^C accumulation (nmol ^13^C equivalents g^‐1^ FW h^‐1^) as a proxy in wild‐type (WT) rice and three transgenic lines expressing enzymes of the C_4_ metabolic pathway during ^13^CO_2_‐pulse labelling.
**Table S4.** Metabolite amounts of wild‐type (WT) rice and three transgenic lines expressing enzymes of the C_4_ metabolic pathway.
**Table S5.** Genes differentially regulated in all three transgenic *O. sativa* lines expressing the enzymes of C_4_ metabolic pathway.
**Table S6.** Primers used for RT‐PCR.
**Method S1.** Generation of transgenic rice plants.
**Method S2.** ^13^CO_2_ labelling and quenching procedure.
**Method S3.** Immunolocalization of C_4_ enzymes on leaf sections.
**Method S4.** Leaf gas‐exchange analysis.

**Data S1.** (separate file). Isotopomer and metabolite amounts, ^13^C enrichments and relative isotopomer abundances in wild‐type (WT) rice and three transgenic lines expressing enzymes of the C_4_ metabolic pathway.

**Data S2.** (separate file). The gene read counts obtained by RNA sequencing for wild‐type (WT) and transgenic rice lines expressing the enzymes of the C_4_ metabolic pathway.
