# Supplementary material for: Installation of C4 photosynthetic pathway enzymes in rice using a single construct
Source: Plant Biotechnol J. 2020 Oct 27;19(3):575–88. doi: 10.1111/pbi.13487 (PMC7955876; doi:10.1111/pbi.13487)
Supplement: Supplementary file 1 — Figure S1. Confocal micrographs of C4 enzymes localization on leaf cross‐sections of Z. mays and O. sativa wild‐type (WT) and three transgenic O. sativa lines expressing C4 metabolic enzymes Figure S2. 13CO2 labelling apparatus and quenching procedure Figure S3. 13C enrichment (%) of wild‐type (WT) rice during (A) 13CO2‐pulse labelling and (B) pulse‐chase labelling Figure S4. Net accumulation of 13C (nmol 13C equivalents g‐1 FW) during 13CO2‐pulse labelling of wild‐type (WT) rice and three transgenic lines expressing enzymes of the C4 metabolic pathway Figure S5. Metabolite amounts (nmol g‐1 FW) of wild‐type (WT) rice and three transgenic lines expressing enzymes of the C4 metabolic pathway Figure S6. Relative labelling of PEP and 3PGA during 13CO2‐pulse labelling of wild‐type (WT) rice, three transgenic lines expressing enzymes of the C4 metabolic pathway, maize and cassava Figure S7. Pulse‐chase labelling of wild‐type (WT) rice and three transgenic lines expressing enzymes of the C4 metabolic pathway Figure S8. Isotopomer distribution (%) during pulse‐chase labelling of wild‐type (WT) rice and three transgenic lines expressing enzymes of the C4 metabolic pathway Figure S9. Isotopomer distribution (%) during 13CO2‐pulse labelling of wild‐type (WT) rice and three transgenic lines expressing enzymes of the C4 metabolic pathway Figure S10. Gas‐exchange and fluorescence analysis of wild‐type (WT) O. sativa and three transgenic lines expressing enzymes of the C4 metabolic pathway during a dark‐to‐light shift Figure S11. Number of differentially regulated genes in the three transgenic O. sativa lines expressing the C4 metabolic pathway compared to WT Figure S12. Constructs used for stable rice transformation Figure S13. RT‐PCR detection of Z. mays gene transcripts in T0 O. sativa lines transformed with the gene construct for C4 metabolic pathway expression Table S1. Summary of C4 enzymes localization from the confocal images on Figure 1c and Figure S1. Table S2. Estimati [file PBI-19-575-s001.docx]

**Supporting Information**


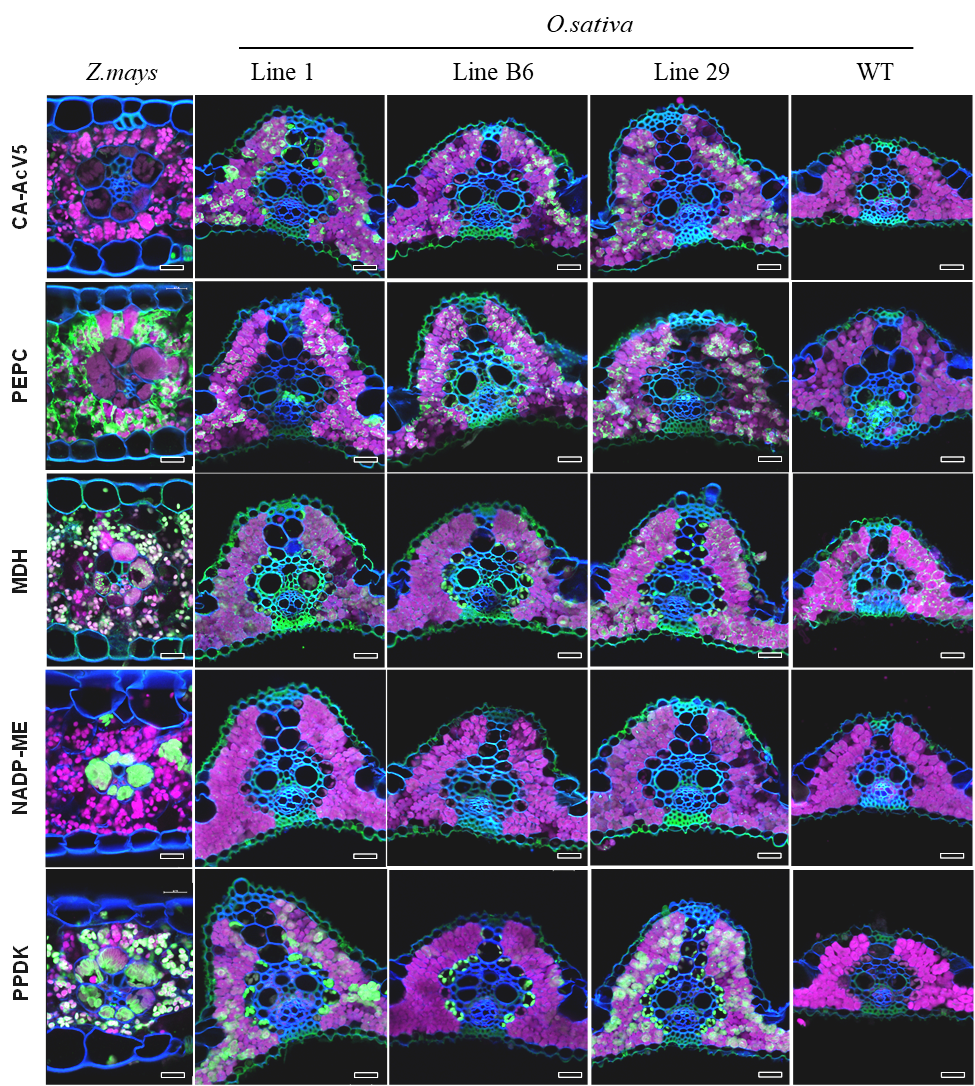


**Fig. S1.** Confocal micrographs of C_4_ enzymes localisation on leaf cross-sections of *Z. mays* and *O. sativa* wild-type (WT) and three transgenic *O. sativa* lines expressing C_4_ metabolic enzymes: CA-AcV5, carbonic anhydrase with AcV5 tag; PEPC, PEP carboxylase; MDH, NADP-malate dehydrogenase; NADP-ME, NADP-dependent malic enzyme; PPDK, pyruvate orthophosphate dikinase. Fluorescence signals are pseudo-coloured: green - protein of interest labelled with secondary antibodies conjugated with AlexaFluor488; magenta - chlorophyll autofluorescence; blue - calcofluor white-stained cell walls. Scale bars = 20 µm.


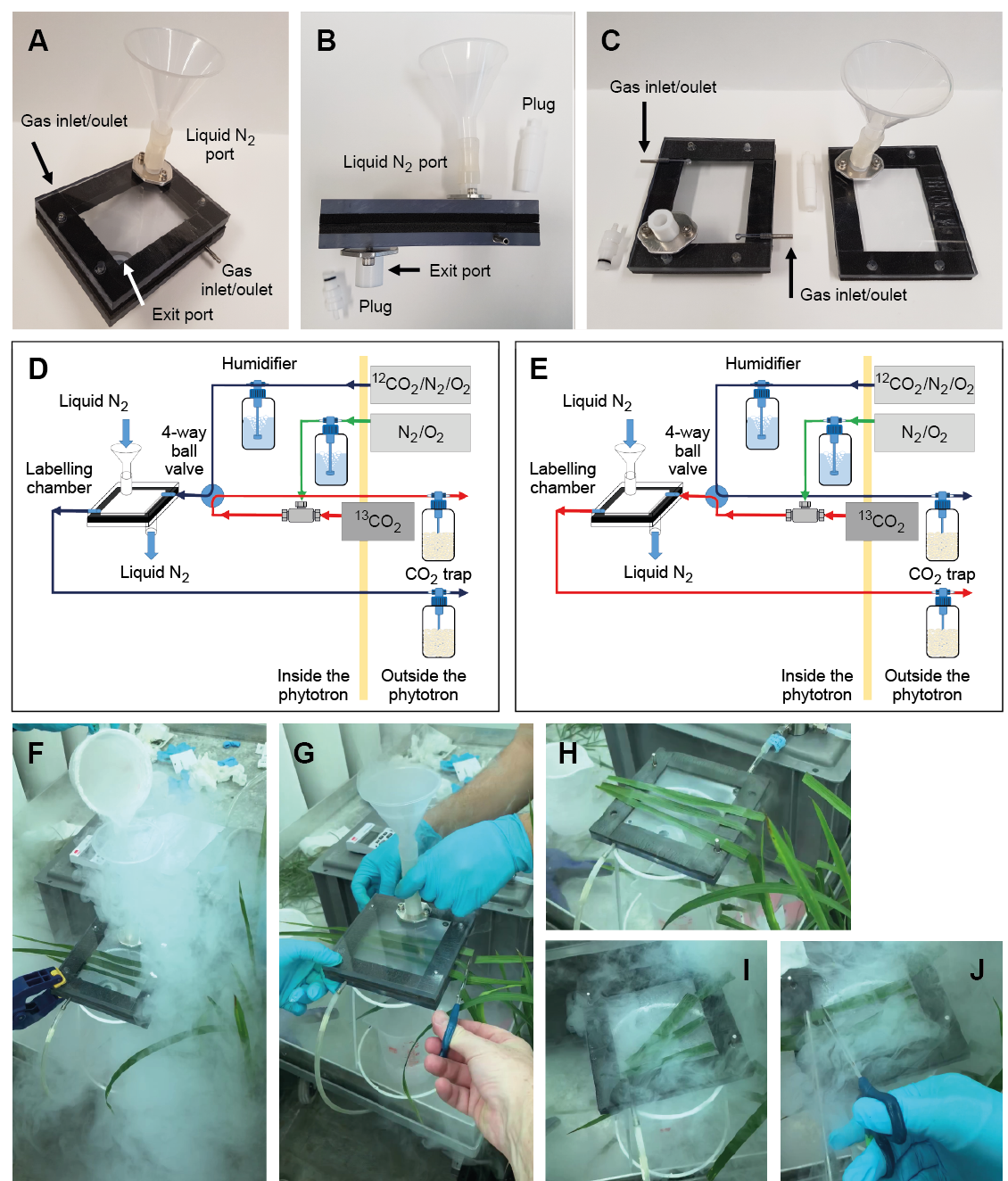


**Fig. S2.** ^13^CO_2_ labelling apparatus and quenching procedure**.** (**A**, **B** and **C**) Custom-made labelling chamber (12 cm x 14.6 cm x 0.6 cm). (**D** and **E**) Schematic representation of the labelling apparatus and gas supplies. (**F**) Quenching of the leaves in the chamber with liquid N_2_. (**G**) Detachment of leaves and removal of external leaf sections before opening the labelling chamber. (**H**) Frozen leaves in opened labelling chamber. (**I** and **J**) Removal of non-illuminated parts of the leaves that were shaded by the rubber gasket while remaining tissue was kept frozen by flooding with liquid N_2_.


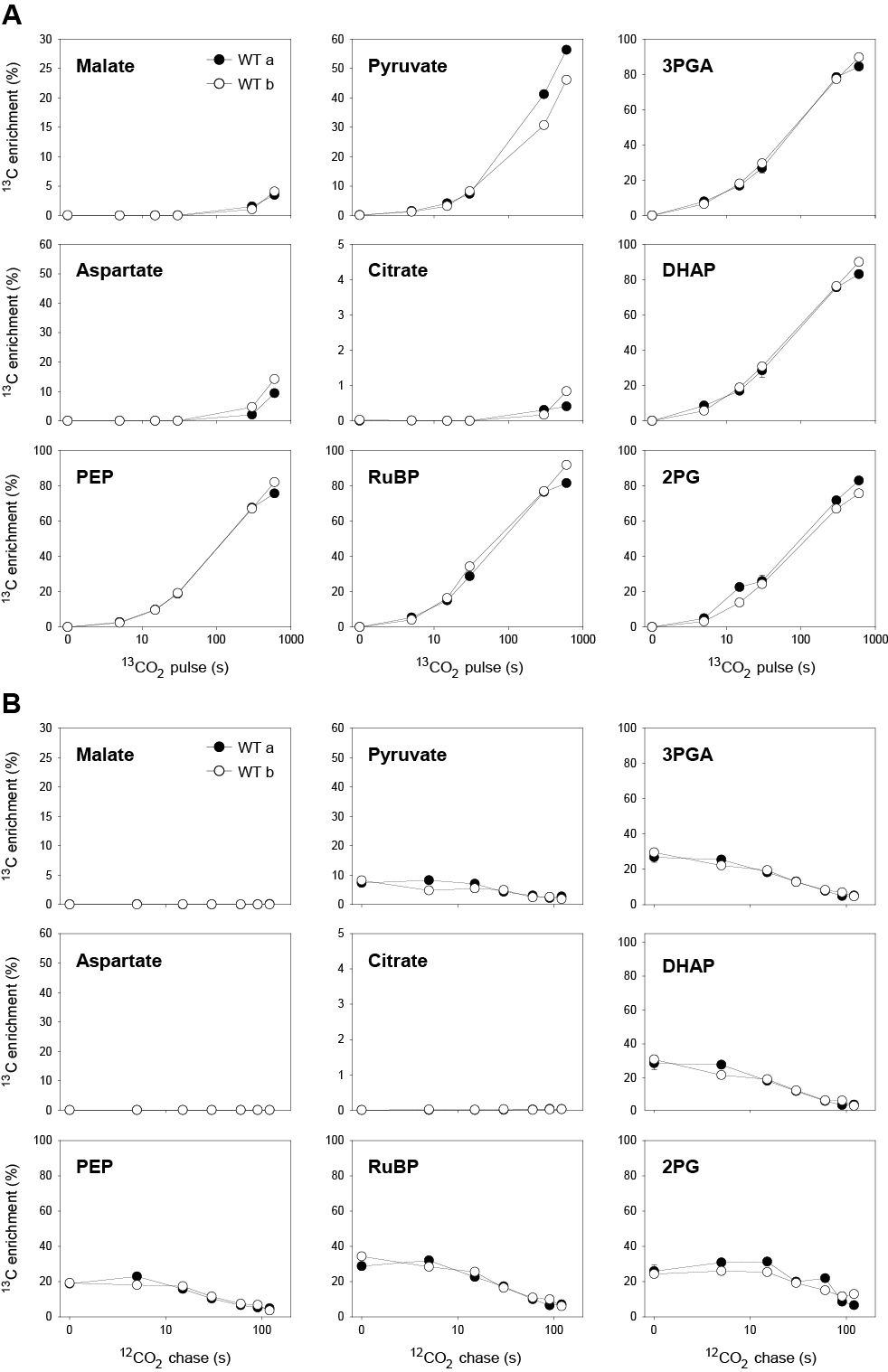


**Fig. S3.**  ^13^C enrichment (%) of wild-type (WT) rice during (**A**) ^13^CO_2_-pulse labelling and (**B**) pulse-chase labelling experiments performed 18 days apart (60/61 DAS for WT a and 79 DAS for WT b). The x-axes show the pulse labelling time on a log scale. Values from WT a 30 s time point are the mean ± SD of 3-4 biological replicates. Values at all other time points are from individual samples or means of two biological replicates. The original data are presented in Data S1.

**
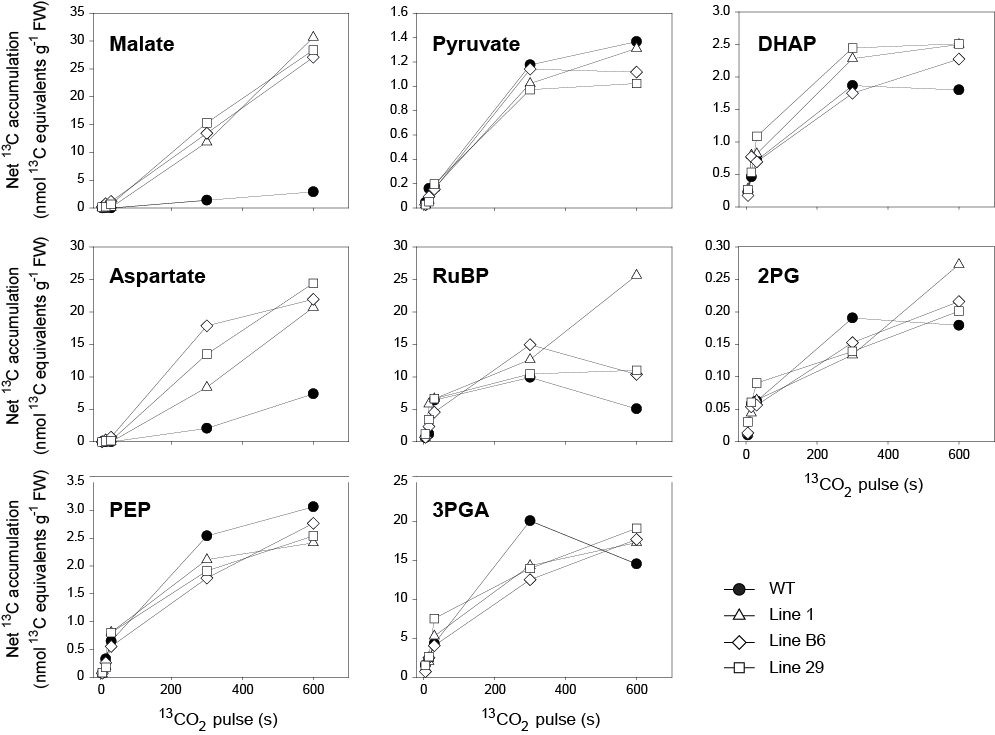
**

**Fig. S4.** Net accumulation of ^13^C (nmol ^13^C equivalents g^-1^ FW) during ^13^CO_2_-pulse labelling of wild-type (WT) rice and three transgenic lines expressing enzymes of the C_4_ metabolic pathway. No estimation was made for citrate due to the extremely low labelling of this metabolite. The original data are presented in Data S1.


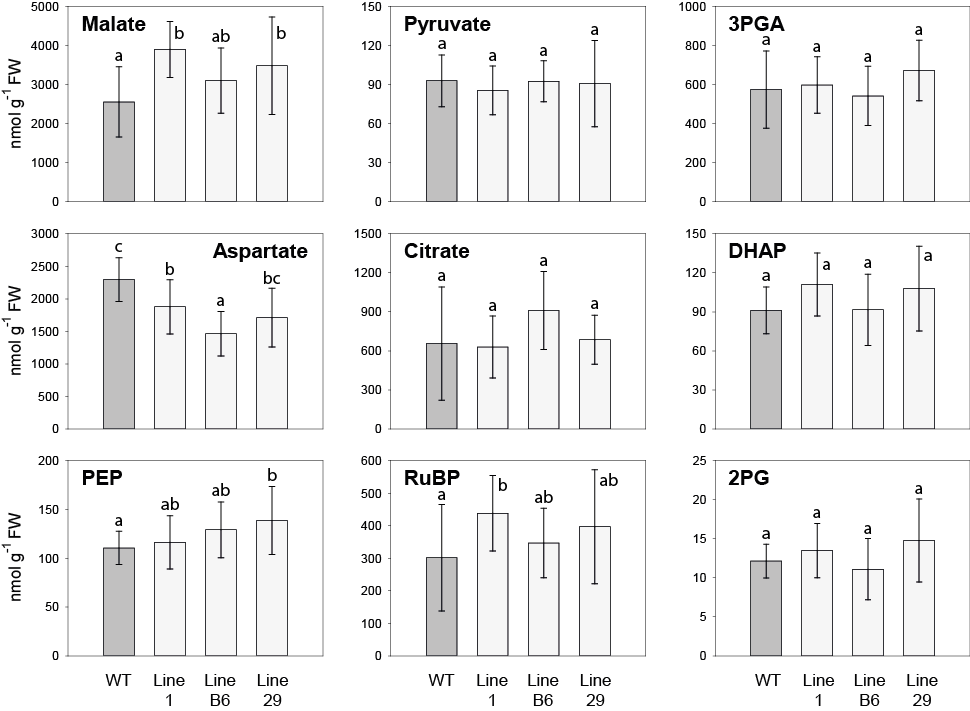


**Fig. S5.** Metabolite amounts (nmol g^-1^ FW) of wild-type (WT) rice and three transgenic lines expressing enzymes of the C_4_ metabolic pathway. Mean ± SD, n = 13 to 17 biological replicates (from both the ^13^CO_2_-pulse labelling and pulse-chase labelling performed at 60 and 61 DAS; samples that were common to both experiments, i.e. 30 s ^13^CO_2_-pulse/0 s chase, were included only once for these calculations). Statistical analysis was performed using one-way ANOVA and different letters indicate significantly different values according to Tukey’s *post-hoc* test (α > 0.05). The original data are presented in Data S1 and Table S2. g FW m^-2^ for WT was 132 ± 11 (n = 10).


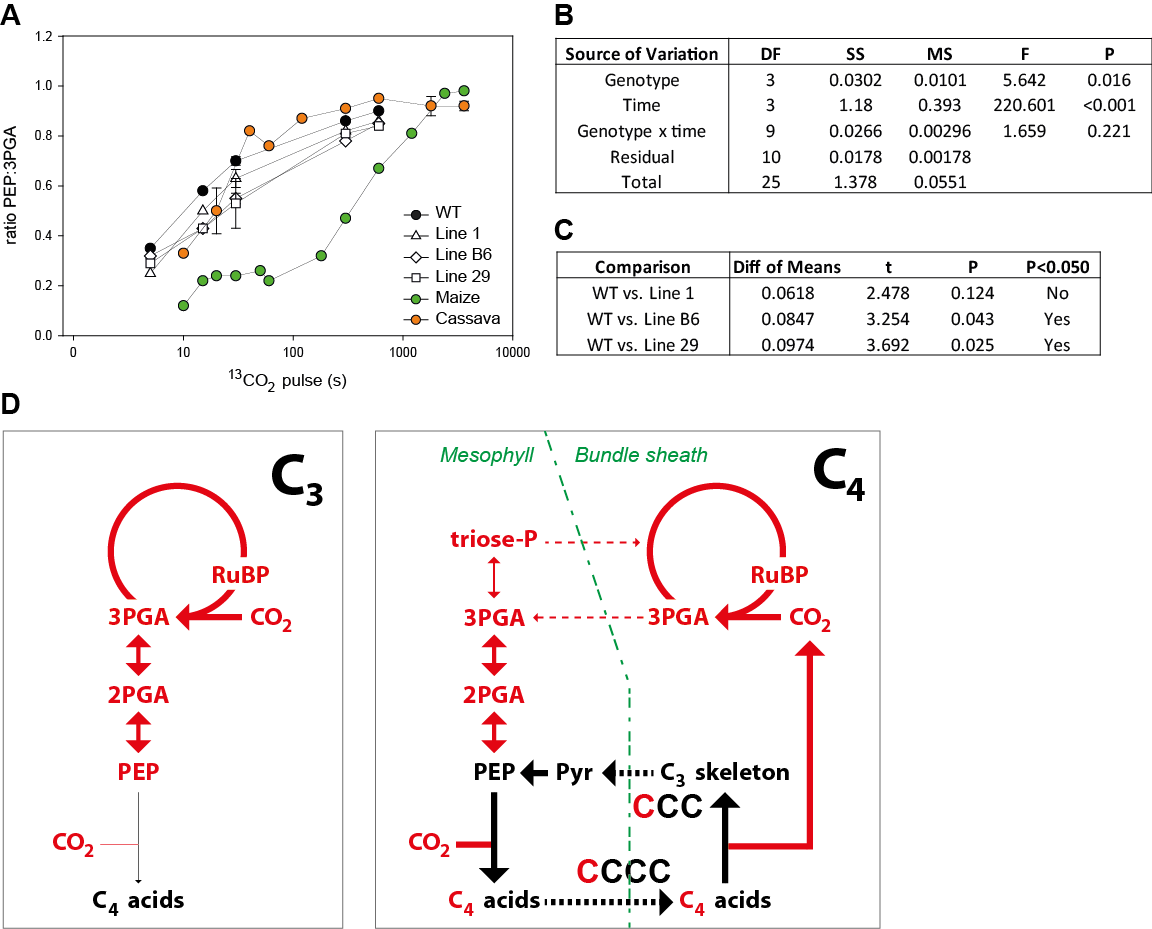


**Fig. S6.** Relative labelling of PEP and 3PGA during ^13^CO_2_-pulse labelling of wild-type (WT) rice, three transgenic lines expressing enzymes of the C_4_ metabolic pathway, maize and cassava. (**A**) Ratio PEP:3PGA calculated from the respective ^13^C enrichments of PEP and 3PGA. The x-axes show the pulse labelling time on a log scale. Values for rice from the 30 s time point are the mean ± SD of 3-4 biological replicates. Values for rice at all other time points are from individual samples or means of two biological replicates. The original data for rice are presented in Data S1. (**B, C**) Statistical analysis. (B) Two-way ANOVA for WT rice and the three transgenic lines based on regressions. Time points 5 to 30 s were used and a time point of 1 s was added with a value of zero, representing unlabelled plants (1 s was chosen based on the gas half-time in the labelling chamber which was 0.35 sec (see Method S2). Regressions were calculated using time expressed on a log_10_ basis. (**C**) All pairwise multiple comparison procedures (Holm-Sidak method) comparing genotypes based on their regressions. (**D**) Comparison of the flow of ^13^C into PEP during the initial phase of a ^13^CO_2_ pulse in rice and maize. Flow of ^13^C is indicated by red arrows and red font, the pre-existing ^12^C pools and fluxes from these pools are denoted by black font and black arrows. The thickness of the arrow indicates the relative flux. Irreversible reactions are indicated by a single arrow, reversible reactions by a double arrow, and intercellular movement of metabolites by dashed arrows. Intercellular compartmentation of C_4_ photosynthesis is shown but, for simplicity, intracellular compartmentation of C_3_ and C_4_ photosynthesis is not shown. The left panel shows rice as an example of a C_3_ plant. ^13^C is rapidly incorporated into and randomized in Calvin-Benson cycle intermediates including 3PGA, and label in 3PGA rapidly moves into 2PGA and PEP via the reversible reactions catalysed by phosphoglyceromutase and enolase, with enrichment in PEP rising almost as rapidly as that in 3PGA (see Fig. 2, Fig. S3, Fig. S4, Fig. S9). Label in C_4_ acids rises slowly due to low activity of PEPC. The right panel shows maize as an example of an NADP-ME C_4_ plant. High PEPC activity in the mesophyll leads to rapid incorporation of ^13^C into the C-1 position of C_4_ acids, which move (e.g. as malate) into the bundle sheath cells where the labelled ^13^C at the C-1 position is released and incorporated into Calvin-Benson cycle intermediates. The unlabelled three-carbon skeleton (e.g. pyruvate) moves back to the mesophyll cells where it is converted to PEP by PPDK. This influx of unlabeled C is equivalent to at least three times the net rate of C fixation, and even more if there is some photorespiration or back leakage of CO_2_ from the bundle sheath to the mesophyll. For comparison, label flow from 3PGA to PEP is about 9-14% of the net rate of C fixation. This large influx of unlabeled C from the C_4_ cycle delays the rise in ^13^C enrichment in PEP. Over time, reversible interconversion of malate and fumarate by fumarase can lead to equilibration between C-1 and C-4 of malate and labelling of C-4, resulting in partial labelling of pyruvate and PEP following decarboxylation of malate. Label might also enter PEP from ^13^C-labelled 3PGA, via phosphoglyceromutase and enolase, as occurs in C_3_ plants.


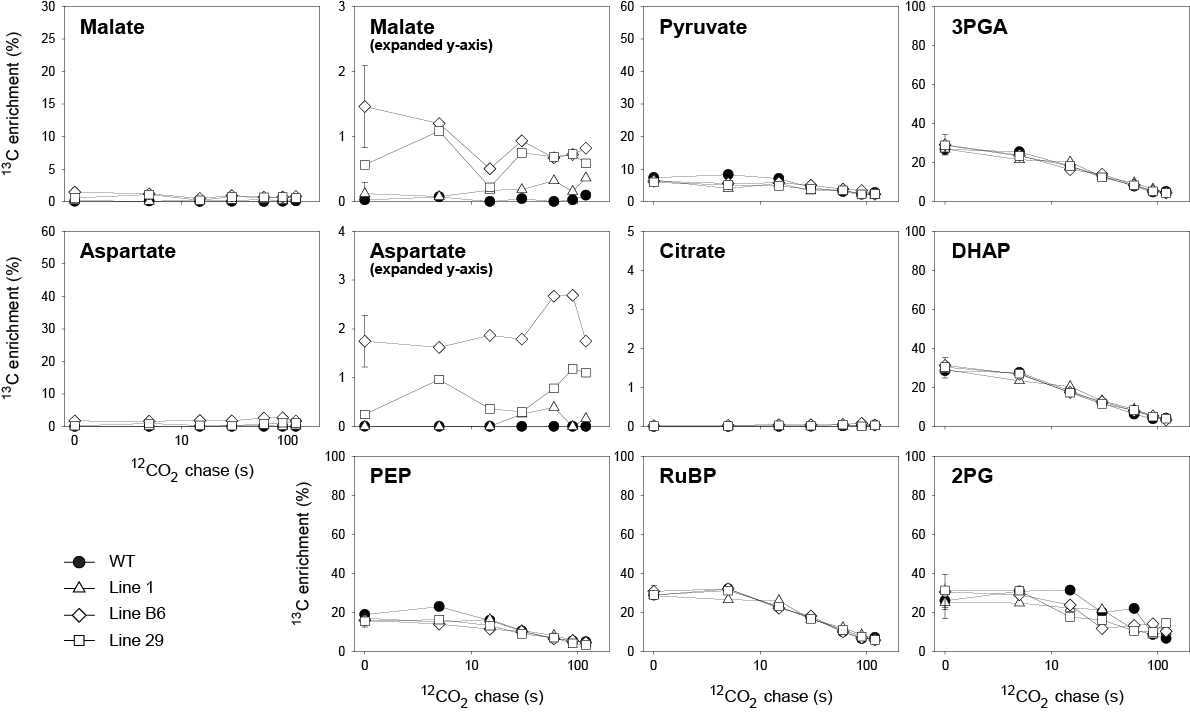


**Fig. S7.** Pulse-chase labelling of wild-type (WT) rice and three transgenic lines expressing enzymes of the C_4_ metabolic pathway. Leaves were pulse labelled with ^13^CO_2_ for 30 s and then sampled at intervals during a chase with ^12^CO_2_ for measurement of ^13^C enrichment (%) of individual metabolites. The x-axes show the time of the chase on a log scale. Values from the 30 s time point are the mean ± SD of 3-4 biological replicates. Values at all other time points are from individual samples. The original data are presented in Data S1.

**A**


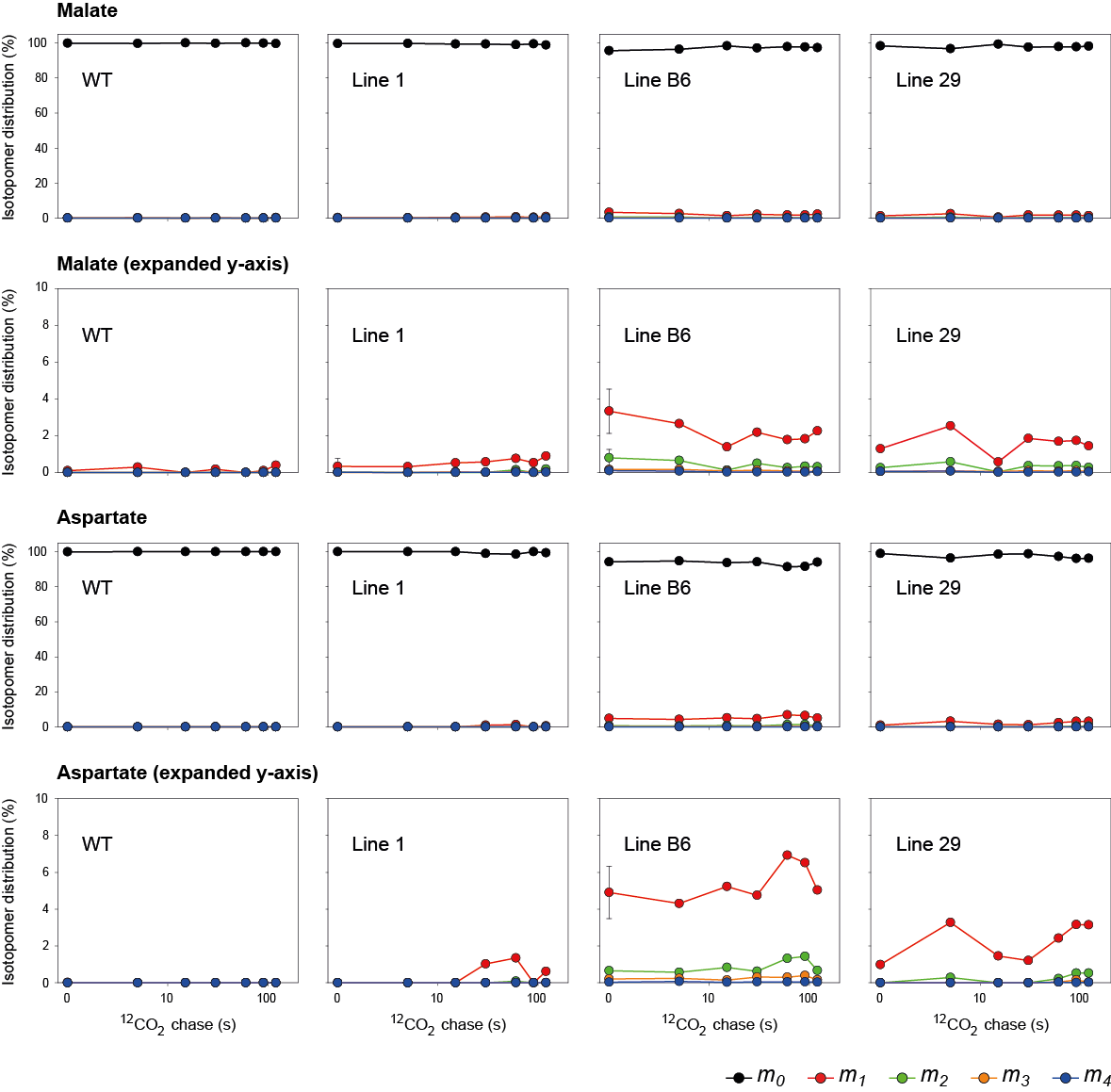


**Fig. S8.** Isotopomer distribution (%) during pulse-chase labelling of wild-type (WT) rice and three transgenic lines expressing enzymes of the C_4_ metabolic pathway. (**A**) Malate and aspartate. The relative abundance of each isotopomer (*m_n_*) for a given metabolite is represented, where *n* is the number of ^13^C atoms incorporated. The x-axes show to the time of the chase on a log scale. Values from the 30 s time point are the mean ± SD of 3-4 biological replicates. Values at all other time points are from individual samples. The original data are presented in Data S1.

**B**

**
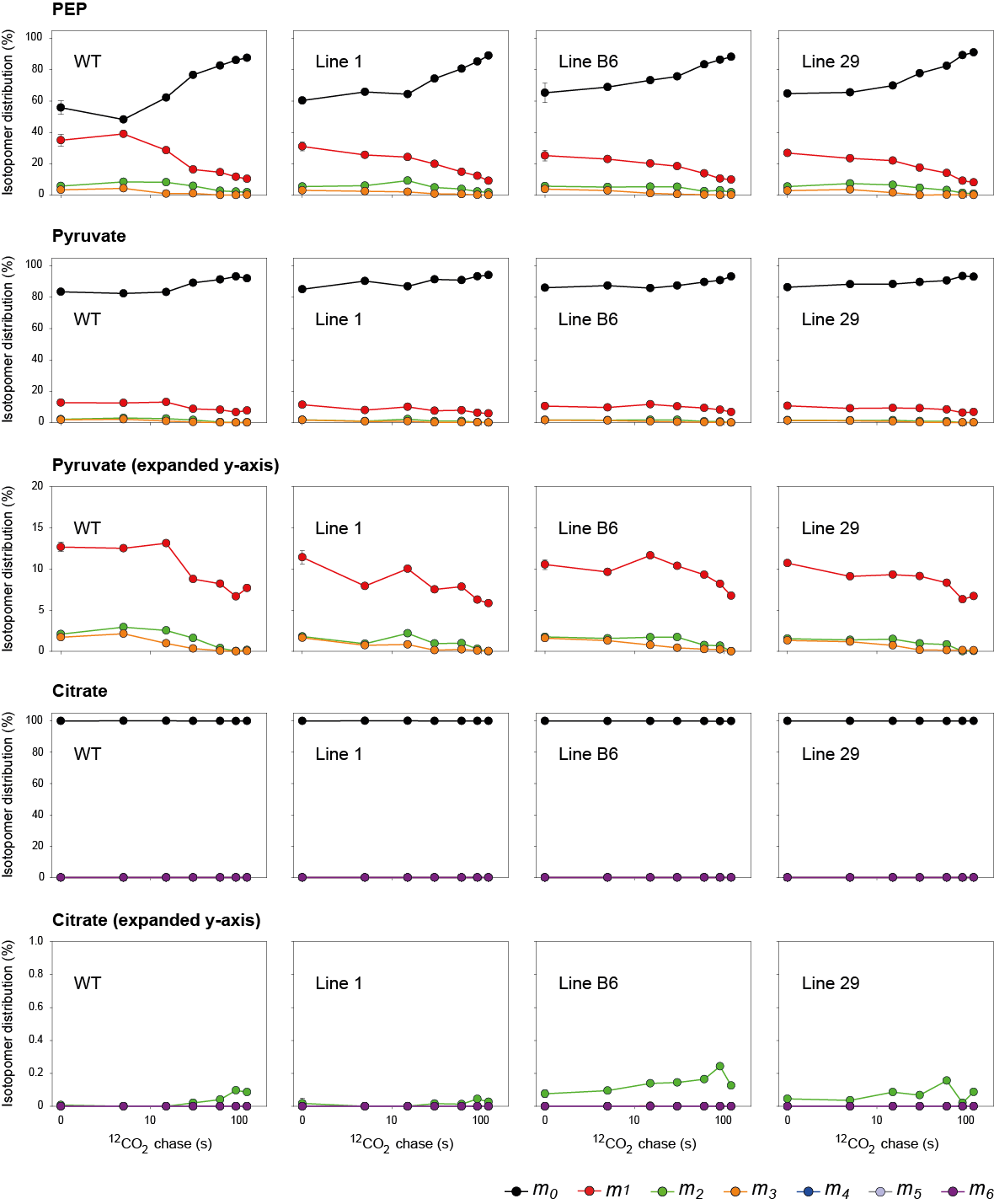
**

**Fig. S8.** Isotopomer distribution (%) during pulse-chase labelling of wild-type (WT) rice and three transgenic lines expressing enzymes of the C_4_ metabolic pathway. (**B**) PEP, pyruvate and citrate. The relative abundance of each isotopomer (*m_n_*) for a given metabolite is represented, where *n* is the number of ^13^C atoms incorporated. The x-axes show to the time of the chase on a log scale. Values from the 30 s time point are the mean ± SD of 3-4 biological replicates. Values at all other time points are from individual samples. The original data are presented in Data S1.

**C**

**
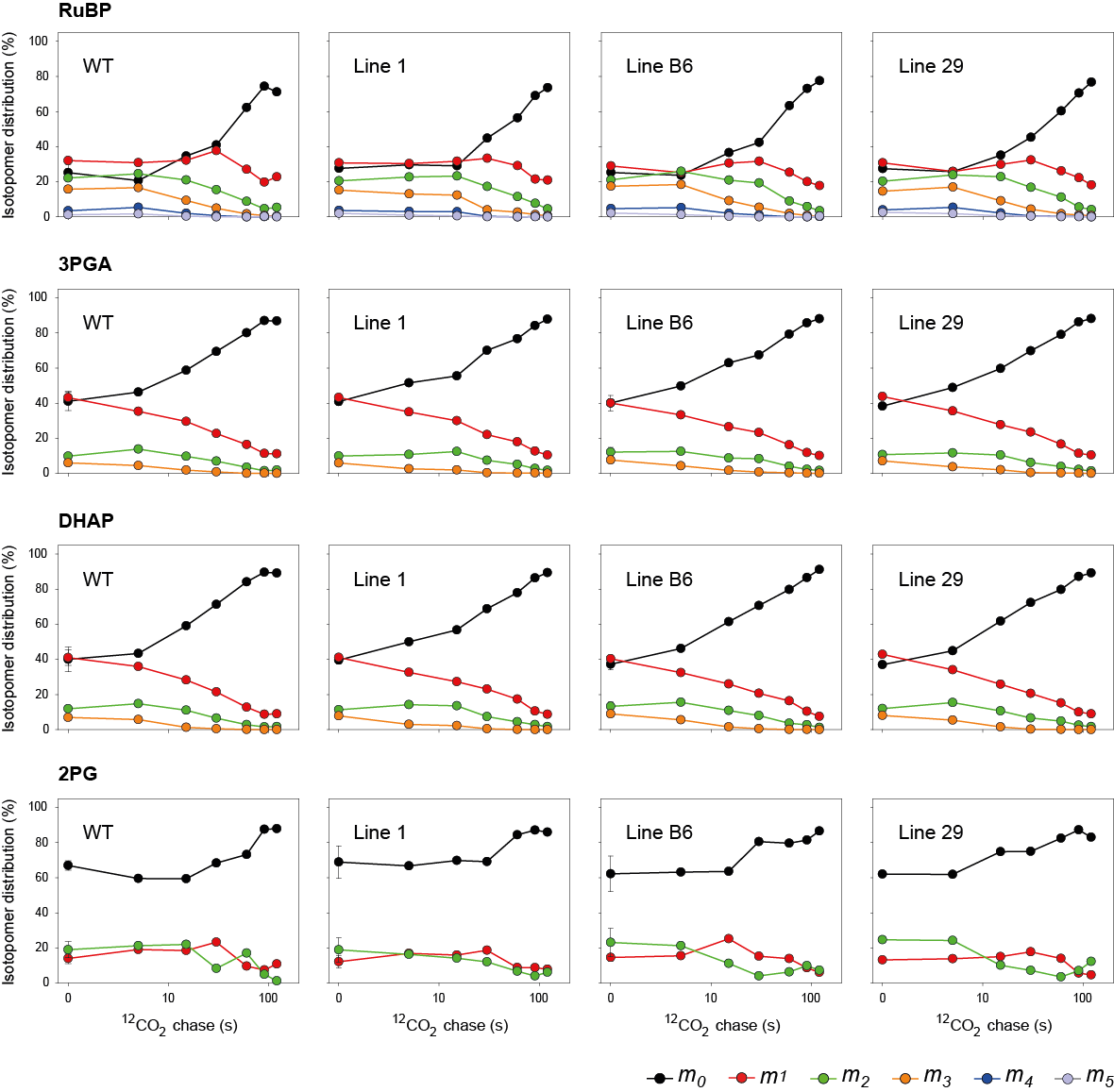
**

**Fig. S8.** Isotopomer distribution (%) during pulse-chase labelling of wild-type (WT) rice and three transgenic lines expressing enzymes of the C_4_ metabolic pathway. (**C**) metabolites involved in the Calvin-Benson cycle and photorespiration. The relative abundance of each isotopomer (*m_n_*) for a given metabolite is represented, where *n* is the number of ^13^C atoms incorporated. The x-axes show to the time of the chase on a log scale. Values from the 30 s time point are the mean ± SD of 3-4 biological replicates. Values at all other time points are from individual samples. The original data are presented in Data S1.

**
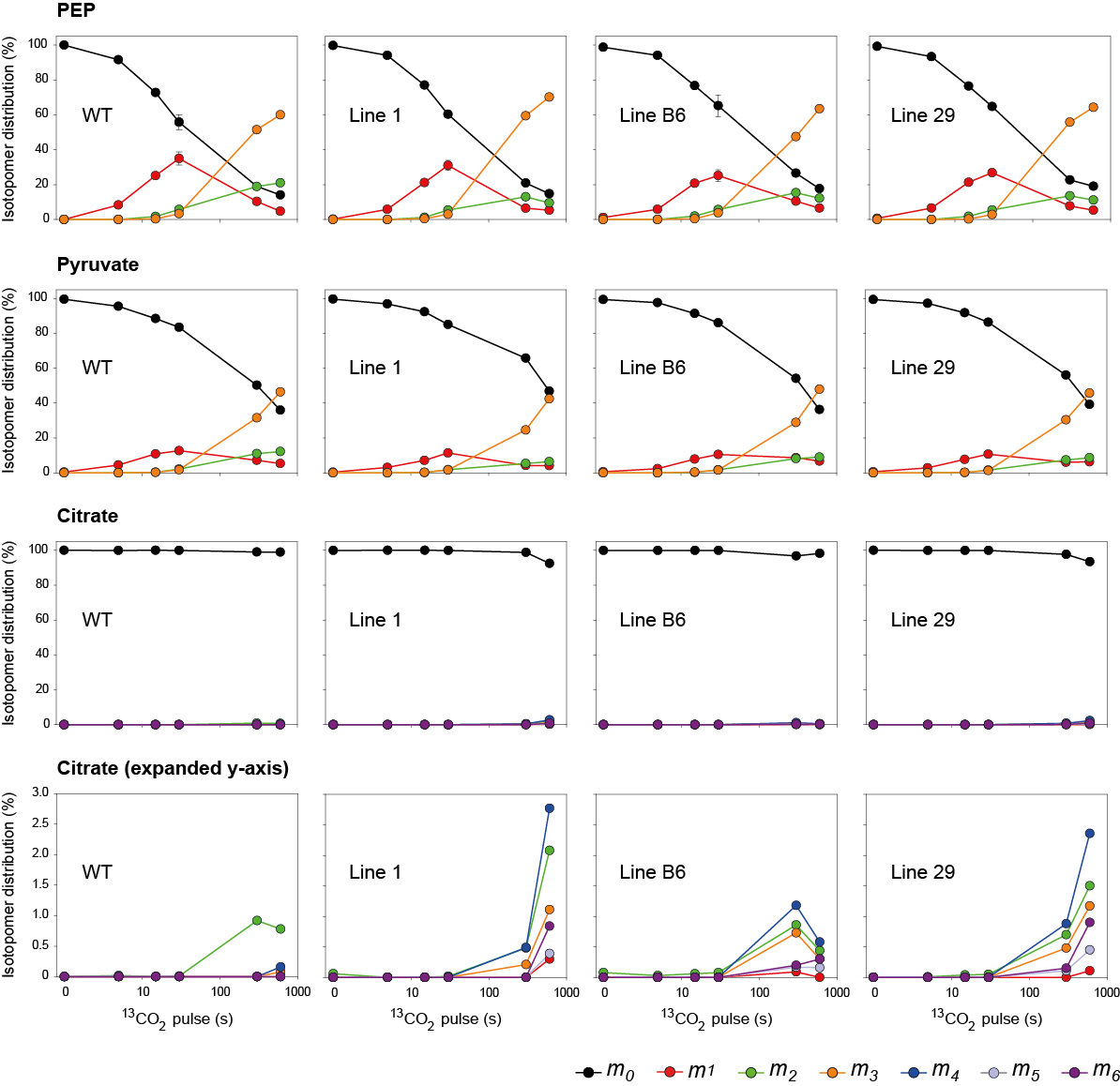
A**

**Fig. S9.** Isotopomer distribution (%) during ^13^CO_2_-pulse labelling of wild-type (WT) rice and three transgenic lines expressing enzymes of the C_4_ metabolic pathway. (**A**) PEP, pyruvate and citrate. The relative abundance of each isotopomer (*m_n_*) for a given metabolite is represented, where *n* is the number of ^13^C atoms incorporated. The x-axes show the pulse labelling time on a log scale. Values from the 30 s time point are the mean ± SD of 3-4 biological replicates. Values at all other time points are from individual samples or means of two biological replicates. The original data are presented in Data S1.

**B**


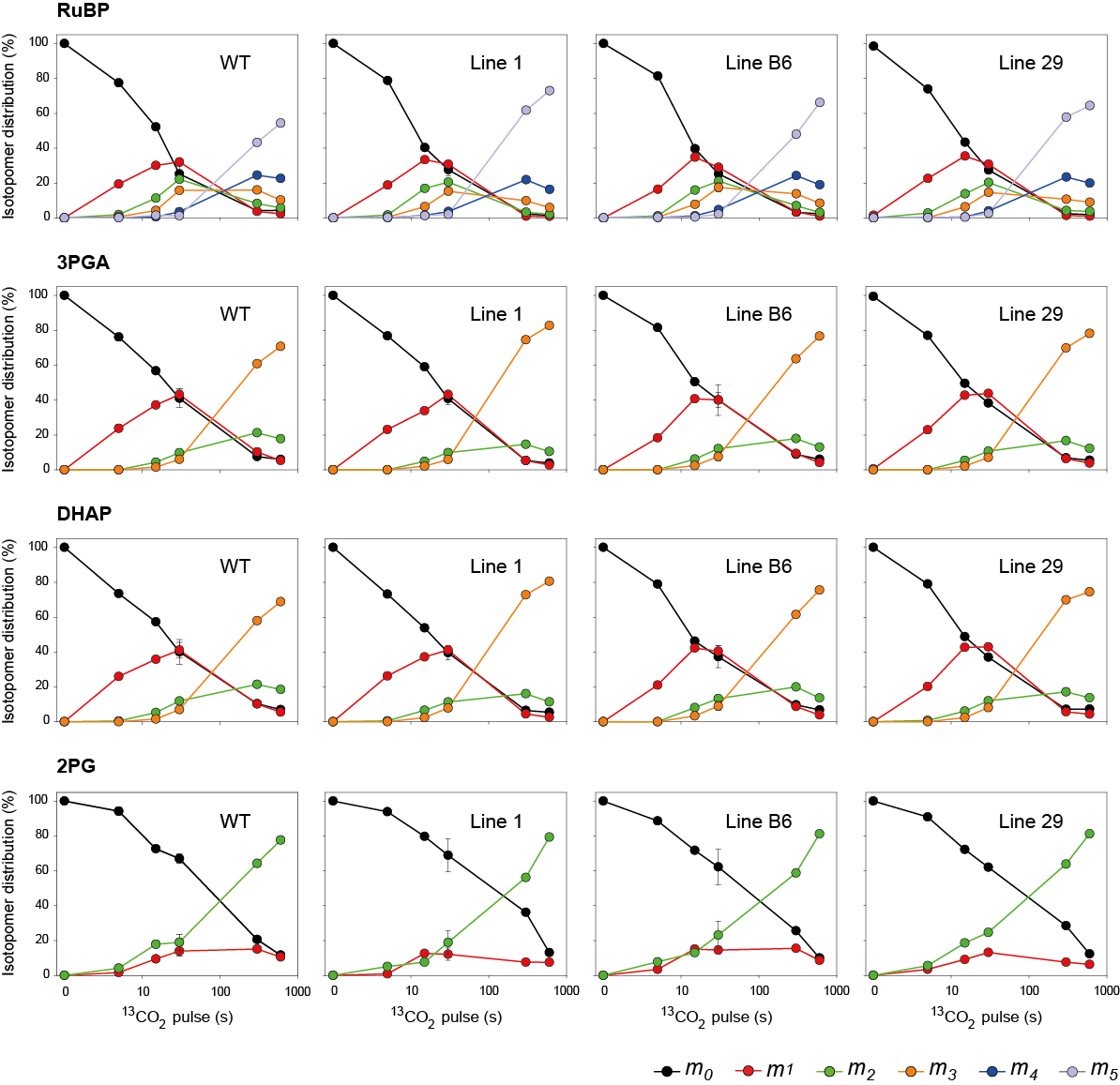


**Fig. S9.** Isotopomer distribution (%) during ^13^CO_2_-pulse labelling of wild-type (WT) rice and three transgenic lines expressing enzymes of the C_4_ metabolic pathway. (**B**) metabolites involved in the Calvin-Benson cycle and photorespiration. The relative abundance of each isotopomer (*m_n_*) for a given metabolite is represented, where *n* is the number of ^13^C atoms incorporated. The x-axes show the pulse labelling time on a log scale. Values from the 30 s time point are the mean ± SD of 3-4 biological replicates. Values at all other time points are from individual samples or means of two biological replicates. The original data are presented in Data S1.

**Fig. S10**. Gas-exchange and fluorescence analysis of wild-type (WT) *O. sativa* and three transgenic lines expressing enzymes of the C_4_ metabolic pathway during a dark-to-light shift. Measurements were done on 40 min dark-adapted leaves, at PPFD of 1000 µmol m^-2^ s^-1^ and ambient *p*O_2_ (20 kPa). A*,* net CO_2_ assimilation rate; g_s_H2O_, leaf stomatal conductance to water vapour; Φ_PSII_, photochemical yield of Photosystem II; NPQ, non-photochemical quenching. Mean ± SE, n = 5 biological replicates for WT, n = 4 otherwise (α > 0.05). No significant difference was found between the transgenic lines and WT at 6, 12, 18 and 24 min after the transition, as evaluated by one-way ANOVA and Tukey’s *post hoc* test (α = 0.05).


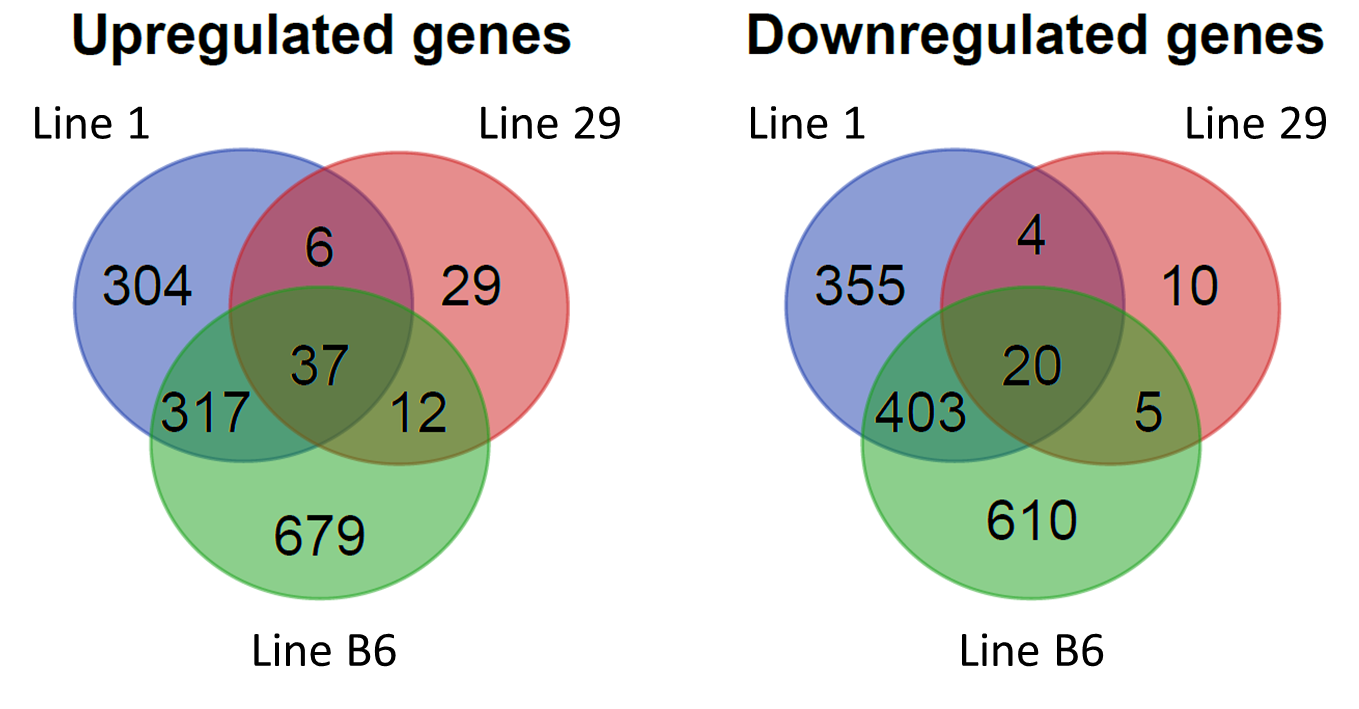


**Fig. S11**. Number of differentially regulated genes in the three transgenic *O. sativa* lines expressing the C_4_ metabolic pathway compared to WT. The original data are presented in Data S2.


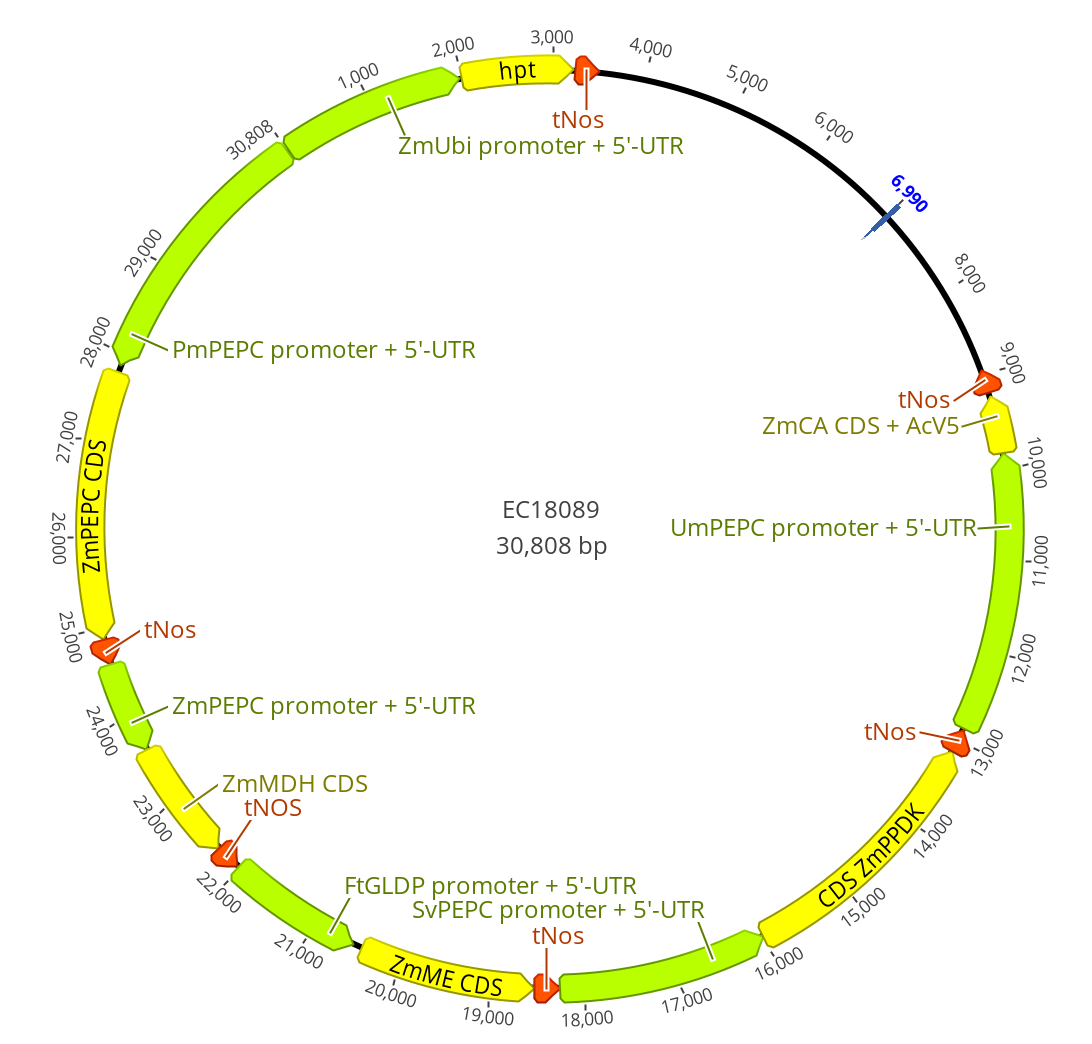


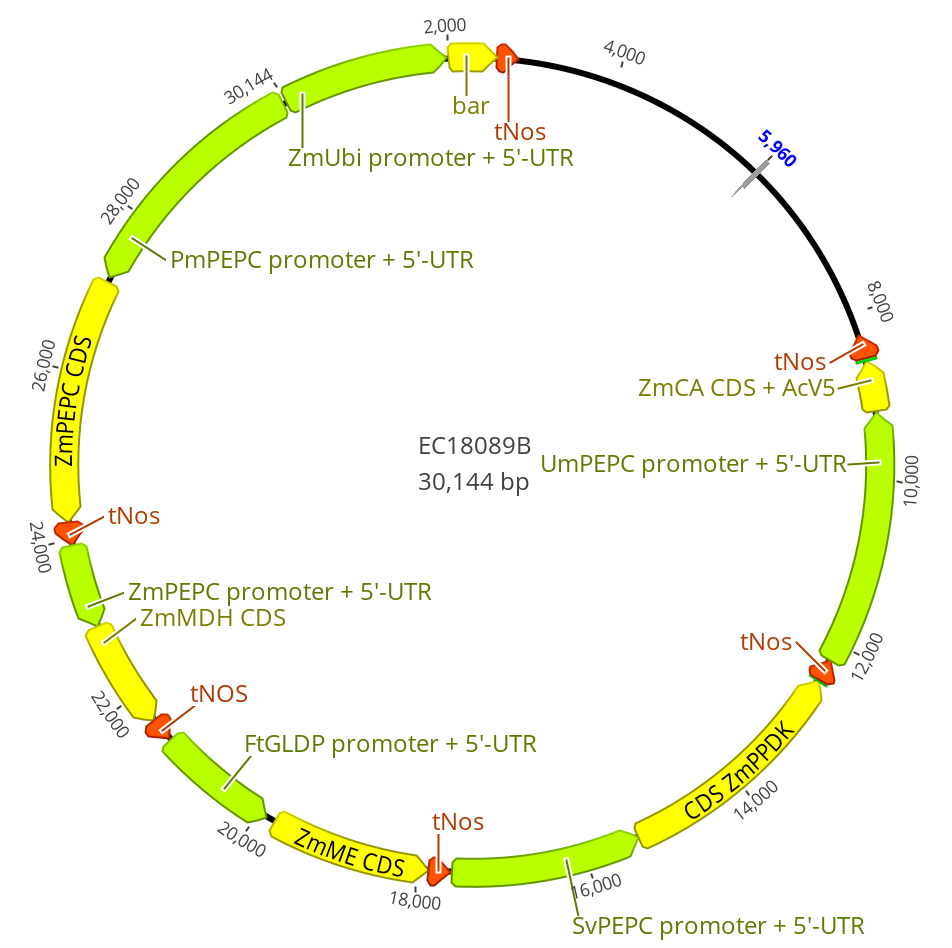


**Fig. S12**. Constructs used for stable rice transformation.


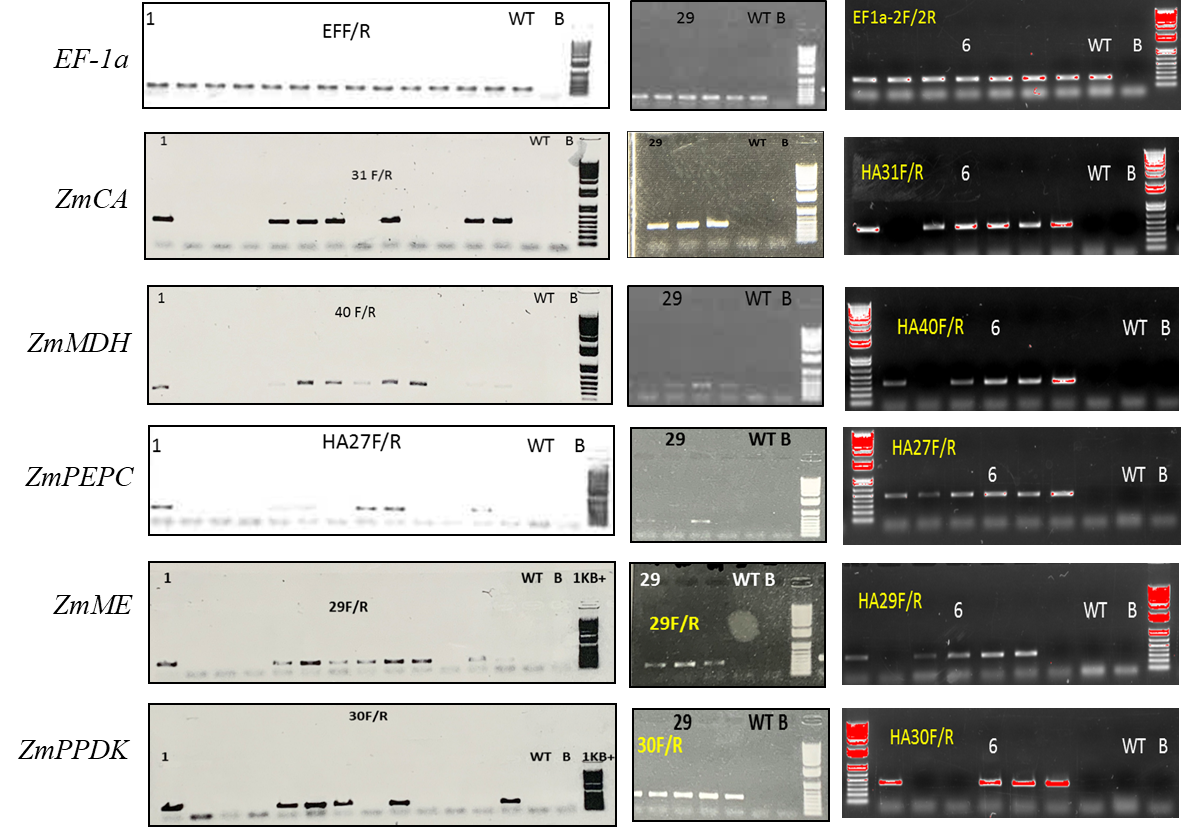


**Fig. S13**. RT-PCR detection of *Z. mays* gene transcripts in T_0_ *O. sativa* lines transformed with the gene construct for C_4_ metabolic pathway expression. Three T_0_ lines selected to be used in this work are marked with numbers: 1, 29 and 6. WT, wild-type *O. sativa*; B, blank negative control, 1KB+, DNA ladder. Primer sequences used for PCR are listed in Table S6. EF-1a, *O. sativa* elongation factor 1 alpha; *ZmCA*, *Z. mays* carbonic anhydrase; *ZmPEPC*, *Z. mays* PEP carboxylase; *ZmMDH*, *Z. mays* NADPH-malate dehydrogenase; *ZmME*, *Z. mays* NADP-dependent malic enzyme; *ZmPPDK*, *Z. mays* pyruvate orthophosphate dikinase.

**Table S1**. Summary of C_4_ enzymes localisation from the confocal images on Fig. 1c and Fig. S1. M, mesophyll; BS, bundle sheath cells; V, vascular bundle. PEPC, PEP carboxylase; PPDK, pyruvate orthophosphate dikinase; NADP-ME, NADP-dependent malic enzyme; MDH, NADP-malate dehydrogenase; CA-AcV5, carbonic anhydrase with AcV5 tag.

| **Genotype** | **PEPC** | **PPDK** | **NADP-ME** | **MDH** | **CA-AcV5** |
| --- | --- | --- | --- | --- | --- |
| *O. sativa* WT | Cytosolic in V | No signal | No signal | Mitochondrial in M | No signal |
| *Z. mays* | Cytosolic in M | Chloroplastic in M and BS | Chloroplastic in BS | Mitochondrial in BS  Chloroplastic in M | No signal |
| Line 1 | Cytosolic in M, BS and V | Chloroplastic in M and BS | Mitochondrial in M | Chloroplastic in M and BS Mitochondrial in M | Cytosolic in M and BS |
| Line 29 | Cytosolic in M and V | Chloroplastic in M and BS | Mitochondrial in M | Chloroplastic in M and BS  Mitochondrial in M | Cytosolic in M |
| Line B6 | Cytosolic in M and V | Chloroplastic in M and BS | No signal | Chloroplastic in M and BS  Mitochondrial in M | Cytosolic in M and BS |

**Table S2**. Estimation of ^13^C enrichment half times in different metabolites in wild-type (WT) rice and three transgenic lines expressing enzymes of the C_4_ metabolic pathway during ^13^CO_2_-pulse labelling. To estimate the half-time (S_0.5_, expressed in s), a linear transformation (x-axis = log_10_ ^13^C pulse duration (s); y-axis = log_10_ (^13^C enrichment / (1 - ^13^C enrichment)) was done with the ^13^C enrichment data, omitting the zero timepoint, as metabolites were not labelled at this time point. The slope and intercept are also provided. For malate, two options were used for calculation: using time points from 5 to 600 s or time points from 5 to 300 s as the data for line B6 at 600 s may be an outlier. For aspartate, time points with no labelling were excluded. No estimation of ^13^C enrichment half-time was made for citrate due to the low labelling of this metabolite. The original data are provided in Data S1.

| Metabolite | Parameter | **WT** | **Line 1** | **Line B6** | **Line 29** |
| --- | --- | --- | --- | --- | --- |
| Malate | Slope | 1.2 | 1.3 | 1.0 | 1.3 |
|  | Intercept | -4.8 | -4.5 | -3.3 | -4.1 |
|  | **S_0.5_ (s)** | **12267** | **2389** | **2897** | **1457** |
| Malate (excluding 600 s) | Slope | 1.1 | 1.3 | 1.0 | 1.2 |
|  | Intercept | -4.7 | -4.4 | -3.3 | -4.1 |
|  | **S_0.5_ (s)** | **20155** | **3166** | **2374** | **1798** |
| Aspartate (excluding time points if y=0) | Slope | 2.6 | 1.6 | 1.6 | 1.7 |
|  | Intercept | -8.3 | -4.8 | -4.3 | -4.8 |
|  | **S_0.5_ (s)** | **1358** | **922** | **531** | **692** |
| PEP | Slope | 1.0 | 1.1 | 1.0 | 1.0 |
|  | Intercept | -2.1 | -2.4 | -2.3 | -2.3 |
|  | **S_0.5_ (s)** | **157** | **152** | **190** | **175** |
| Pyruvate | Slope | 0.9 | 0.9 | 1.0 | 1.0 |
|  | Intercept | -2.5 | -2.6 | -2.7 | -2.7 |
|  | **S_0.5_ (s)** | **448** | **678** | **465** | **504** |
| RuBP | Slope | 0.9 | 1.1 | 1.0 | 1.0 |
|  | Intercept | -1.8 | -2.0 | -1.9 | -1.9 |
|  | **S_0.5_ (s)** | **97** | **64** | **78** | **71** |
| 3PGA | Slope | 0.9 | 1.0 | 0.9 | 1.0 |
|  | Intercept | -1.7 | -1.9 | -1.8 | -1.8 |
|  | **S_0.5_ (s)** | **83** | **63** | **77** | **68** |
| DHAP | Slope | 0.8 | 1.0 | 0.9 | 0.9 |
|  | Intercept | -1.6 | -1.7 | -1.7 | -1.7 |
|  | **S_0.5_ (s)** | **87** | **62** | **74** | **71** |
| 2PG | Slope | 0.9 | 0.9 | 0.8 | 0.8 |
|  | Intercept | -1.8 | -1.8 | -1.5 | -1.6 |
|  | **S_0.5_ (s)** | **97** | **125** | **86** | **88** |

**Table S3.** Estimation of minimum ^13^C fluxes using slopes of ^13^C accumulation (nmol ^13^C equivalents g^-1^ FW h^-1^) as a proxy in wild-type (WT) rice and three transgenic lines expressing enzymes of the C_4_ metabolic pathway during ^13^CO_2_-pulse labelling. To estimate ^13^C fluxes, fractional ^13^C enrichment for a given metabolite at a given time was multiplied by the number of C atoms in the molecule (*n*) and the metabolite amount using amounts determined in each sample (y = ^13^C enrichment**n**amounts versus time). As malate and aspartate exhibited near-linear accumulation with time (Fig. S4), slopes were calculated from the entire time kinetic. For malate, two options were used for calculation: using time points from 5 to 600 s or time points from 5 to 300 s as the data for line B6 at 600 s may be an outlier. For aspartate, time points with no labelling were excluded. For the reminding metabolites, which had non-linear kinetics for ^13^C accumulation (Fig. S4), a Hanes linear transformation $\frac{x}{y}$ ($=\frac{\mathrm{time}}{13C enrichment*amount}$) versus x ($=$time) was performed. Slopes and intercepts were calculated and used to determine Y_max_ ($=\frac{1}{\mathrm{slope}}$) and X_0.5_ ($=\frac{\mathrm{intercept}}{\mathrm{Ymax}}$). Using the equation $y=\frac{Ymax * x}{X0.5+x}$, y_1_ and y_2_ values were calculated with x_1_= 5 and x_2_= 10, respectively, and the initial slopes ($=\frac{y2-y1}{x2-x1}$) were determined. No estimation of ^13^C fluxes was made for citrate due to the extremely low labelling of this metabolite. The calculation steps are provided in Data S1.

|  | **Rate of ^13^C accumulation (nmol ^13^C equivalents g^-1^ FW h^-1^)** | | | |
| --- | --- | --- | --- | --- |
| Metabolite | **WT** | **Line 1** | **Line B6** | **Line 29** |
| Malate | 18 | 184 | 162 | 175 |
| Malate (excluding 600 s) | 17 | 148 | 161 | 190 |
| Aspartate (removing time points where y=0) | 47 | 125 | 144 | 153 |
| PEP | 68 | 57 | 58 | 60 |
| Pyruvate | 30 | 18 | 22 | 19 |
| RuBP | 705 | 601 | 704 | 827 |
| 3PGA | 814 | 623 | 502 | 757 |
| DHAP | 122 | 147 | 108 | 151 |
| 2PG | 10 | 7 | 8 | 11 |

**Table S4.** Metabolite amounts (expressed as nmol g^-1^ FW in **A** and as µmol m^-2^ in **B**) of wild-type (WT) rice and three transgenic lines expressing enzymes of the C_4_ metabolic pathway. For conversion between both units, g FW m^-2^ was determined for WT (n = 10; 132 ± 11 g FW m^-2^). Mean ± SD, n = 13 to 17 biological replicates (from both the ^13^CO_2_-pulse labelling and pulse-chase labelling performed at 60 and 61 DAS; samples that were common to both experiments, i.e. 30 s ^13^CO_2_-pulse/0 s chase, were included only once for these calculations). The data expressed as nmol g^-1^ FW are plotted in Fig. S5, where statistical analysis is also provided. The original data are presented in Data S1.

**A**

| Metabolite (nmol g^-1^ FW) | **WT** | **Line 1** | **Line B6** | **Line 29** |
| --- | --- | --- | --- | --- |
| Malate | 2557 ± 898 | 3901 ± 716 | 3100 ± 834 | 3481 ± 1249 |
| Aspartate | 2297 ± 336 | 1879 ± 417 | 1465 ± 343 | 1712 ± 451 |
| PEP | 111 ± 17 | 116 ± 27 | 129 ± 29 | 139 ± 35 |
| Pyruvate | 93 ± 20 | 85 ± 19 | 93 ± 16 | 91 ± 33 |
| Citrate | 657 ± 435 | 631 ± 237 | 911 ± 300 | 686 ± 188 |
| RuBP | 302 ± 164 | 438 ± 116 | 347 ± 106 | 397 ± 175 |
| 3PGA | 574 ± 198 | 598 ± 145 | 542 ± 152 | 672 ± 155 |
| DHAP | 91 ± 18 | 111 ± 24 | 92 ± 27 | 108 ± 33 |
| 2PG | 12 ± 2 | 14 ± 3 | 11 ± 4 | 15 ± 5 |

**B**

| Metabolite (µmol m^-2^) | **WT** | **Line 1** | **Line B6** | **Line 29** |
| --- | --- | --- | --- | --- |
| Malate | 338 ± 119 | 515 ± 95 | 410 ± 110 | 460 ± 165 |
| Aspartate | 304 ± 44 | 248 ± 55 | 194 ± 45 | 226 ± 60 |
| PEP | 15 ± 2 | 15 ± 4 | 17 ± 4 | 18 ± 5 |
| Pyruvate | 12 ± 3 | 11 ± 2 | 12 ± 2 | 12 ± 4 |
| Citrate | 87 ± 57 | 83 ± 31 | 120 ± 40 | 91 ± 25 |
| RuBP | 40 ± 22 | 58 ± 15 | 46 ± 14 | 53 ± 23 |
| 3PGA | 76 ± 26 | 79 ± 19 | 72 ± 20 | 89 ± 21 |
| DHAP | 12 ± 2 | 15 ± 3 | 12 ± 4 | 14 ± 4 |
| 2PG | 1.6 ± 0.3 | 1.8 ± 0.5 | 1.5 ± 1 | 2 ± 1 |

**Table S5**. Genes differentially regulated in all three transgenic *O. sativa* lines expressing the enzymes of C_4_ metabolic pathway compared to wild-type (WT) *O. sativa* and possible functions of their orthologues in *Arabidopsis thaliana*. NA, not available. Average Log2fold values for gene counts (WT versus transgenic plants) are provided for each line. The original data are presented in Data S2.

| **Upregulated genes** | **Arabidopsis orthologs** | **Function** | **Line 1** | **Line 29** | **Line B6** |
| --- | --- | --- | --- | --- | --- |
| LOC_Os11g09280 | AT1G77510.1, AT1G21750.1 | Thioredoxin, attenuation of D1 synthesis, modulating photoinhibition in a light-regulated manner | -1.023 | -0.589 | -1.272 |
| LOC_Os05g46520 | AT1G80170.1 | Pectin lyase-like superfamily | -1.499 | -0.747 | -1.716 |
| LOC_Os07g35004 | Many |  | -2.182 | -1.161 | -2.420 |
| LOC_Os04g59130 | AT1G76900, AT1G25280, AT1G43640 | Tubby-like proteins, functions unclear | -1.648 | -0.582 | -1.802 |
| LOC_Os01g06550 | AT5G05660 | Involved in the circadian clock and stomatal closure | -1.379 | -0.514 | -1.361 |
| LOC_Os10g18340 | AT1G13930.1, AT2G03440.1 | Nodulin-like, knock-out mutants in Arabidopsis are hypersensitive to salt stress, QTL for circadian phase | -0.765 | -0.469 | -0.944 |
| LOC_Os01g08270 | AT1G80360.1 | Methionine-specific aminotransferase, coordinates both auxin and ethylene biosynthesis | -1.061 | -0.700 | -0.905 |
| LOC_Os07g39450 | NA | Unknown | -7.459 | -5.773 | -7.488 |
| LOC_Os08g37456 | NA | Unknown | -1.924 | -0.776 | -2.067 |
| LOC_Os05g14240 | AT2G25810.1 | TIP4;1, aquaporin, possible nitrogen transporter | -3.551 | -1.538 | -3.825 |
| LOC_Os03g15920 | AT4G37300 | Ortholog of maternal effect embryo arrest 59 | -1.883 | -0.726 | -2.048 |
| LOC_Os02g53400 | AT5G06690.2 | Chloroplast stroma thioredoxin TRX-LIKE 2.1, oxidises redox-regulated proteins in darkness | -1.701 | -0.730 | -2.015 |
| LOC_Os08g01390 | AT1G34260.1 | Signalling PI-kinase | -1.499 | -0.627 | -2.024 |
| LOC_Os11g39310 | NA | Unknown | -1.353 | -1.064 | -1.500 |
| LOC_Os01g35330 | NA | Unknown | -0.990 | -3.763 | -1.060 |
| LOC_Os11g29920 | NA | Unknown | -3.334 | -3.439 | -3.445 |
| LOC_Os07g43560 | Many |  | -0.662 | -0.396 | -0.693 |
| LOC_Os03g01200 | AT2G44980.2 | ASG3, chromatin remodelling helicase | -1.314 | -0.837 | -1.119 |
| LOC_Os11g30210 | NA | Unknown | -2.031 | -1.112 | -2.184 |
| LOC_Os11g41600 | AT5G57340, AT5G67390 | Ras guanine nucleotide exchange factor, glycosyltransferase-like protein | -0.866 | -0.625 | -0.899 |
| LOC_Os11g41160 | AT1G18640 | PHOSPHOSERINE PHOSPHATASE 1, the last step of serine biosynthesis in the chloroplast | -0.598 | -0.735 | -0.760 |
| LOC_Os01g69840 | NA | Unknown | -3.350 | -1.165 | -3.933 |
| LOC_Os01g63410 | AT3G09050.1 | Chloroplast 8-amino-7-oxononanoate synthase (CO2 liberating), biotin metabolism | -1.231 | -0.713 | -1.480 |
| LOC_Os03g38540 | AT2G20830 | Folic acid binding / transferase | -1.463 | -0.659 | -1.494 |
| LOC_Os07g18230 | Many | Receptor kinase | -1.919 | -1.776 | -3.081 |
| LOC_Os01g50622 | AT1G19660, AT1G75380 | BBD2, BBD1, nuclease interacts with JAZ proteins and thus modulates jasmonate signalling | -3.980 | -1.655 | -5.257 |
| LOC_Os06g28050 | NA | Unknown | -3.871 | -1.648 | -4.360 |
| LOC_Os03g01580 | AT4G00440, AT2G45900, AT3G61380, AT1G01695 | TON1 RECRUITING, GPI-anchored adhesin-like protein | -1.903 | -0.880 | -2.245 |
| LOC_Os05g50390 | NA | Unknown | -8.721 | -7.092 | -9.253 |
| LOC_Os11g43700 | Many |  | -1.691 | -2.029 | -1.979 |
| LOC_Os02g45225 | NA | Unknown | -5.181 | -1.607 | -6.014 |
| LOC_Os07g18240 | Many |  | -1.455 | -1.112 | -2.793 |
| LOC_Os01g16620 | NA | Unknown | -2.348 | -1.019 | -2.769 |
| LOC_Os11g42030 | Many | DUF594 | -0.819 | -1.424 | -1.467 |
| LOC_Os07g34570 | AT5G54770.1 | Thiamine biosynthesis, producing the thiazole portion of thiamine (requires pyruvate) | -2.146 | -1.102 | -2.505 |
| LOC_Os05g34490 | NA | Unknown | -7.106 | -6.175 | -7.501 |
| LOC_Os03g15910 | AT3G09085.1 | Small transmembrane protein of unknown function | -1.790 | -0.770 | -1.946 |
| **Downregulated genes** | **Arabidopsis orthologs** | **Function** | **Line 1** | **Line 29** | **Line B6** |
| LOC_Os02g39360 | AT2G31380.1, AT1G06040.1 | Ortholog of BBX24 & BBX25 in Arabidopsis, photomorphogenesis | 1.120 | 0.483 | 1.803 |
| LOC_Os10g42299 | AT5G46800.1 | Ortholog of BOU, putative photorespiratory transporter, mitochondrial | 1.454 | 0.608 | 2.455 |
| LOC_Os03g20380 | AT5G21326 | Protein kinase | 0.857 | 0.574 | 1.524 |
| LOC_Os01g05840 | AT2G37540.1, AT5G02540.1 | Unknown | 2.104 | 0.785 | 3.049 |
| LOC_Os04g48880 | AT5G52570, AT4G25700 | BETA-CAROTENE HYDROXYLASE 2, converts beta-carotene to zeaxanthin | 1.322 | 0.663 | 1.968 |
| LOC_Os02g58100 | AT5G23380.1, AT5G08360.1, AT1G73210.1, AT1G17830.1 | DUF789 | 4.073 | 1.227 | 4.977 |
| LOC_Os03g57660 | AT3G54720.1 | COP2, glutamate carboxypeptidase, promotes photomorphogenesis and an increased level of cytokinin biosynthesis | 0.841 | 0.658 | 1.287 |
| LOC_Os01g16170 | AT2G41050.1, AT4G20100.1 | Unknown transmembrane protein, upregulated when GLK1 is upregulated in Arabidopsis | 2.422 | 0.831 | 3.680 |
| LOC_Os02g58310 | AT3G19900.1 | Unknown chloroplast protein | 2.481 | 0.637 | 4.287 |
| LOC_Os01g62810 | AT3G55580.1, AT3G53830.1 | Ortholog of TCF1 in Arabidopsis, epigenetic regulator that modulates lignin biosynthesis | 3.151 | 0.868 | 4.127 |
| LOC_Os06g40210 | AT3G15760.1, AT1G52565.1 | Unknown cytochrome P450 | 1.551 | 0.664 | 2.072 |
| LOC_Os01g09310 | AT4G20060 | snRNA processing | 0.812 | 0.710 | 1.364 |
| LOC_Os04g45810 | NA | Unknown | 2.638 | 1.002 | 1.801 |
| LOC_Os01g52260 | AT5G56760.1 | Serine acetyltransferase involved in sulphur assimilation | 1.883 | 1.267 | 1.638 |
| LOC_Os07g06800 | AT5G16010.1 | Unknown chloroplast localised dehydrogenase type enzyme | 2.619 | 0.961 | 4.291 |
| LOC_Os03g41330 | AT3G49940, AT5G67420 | LOB-domain proteins involved in nitrogen metabolism and affecting leaf morphogenesis | 1.393 | 0.975 | 2.402 |
| LOC_Os12g38850 | AT3G29180.1, AT5G39430.1 | DUF1336 | 2.265 | 0.940 | 3.448 |
| LOC_Os04g56710 | AT3G07270.1 | GTP cyclohydrolase, first step in folate synthesis | 1.435 | 0.613 | 1.888 |
| LOC_Os03g16700 | AT1G07540.1, AT5G13820.1, AT5G59430.1, AT3G46590.2, AT3G12560.1 | Telomere-binding like proteins | 1.312 | 0.561 | 1.461 |
| LOC_Os08g06110 | AT2G46830.1 | CCA1, circadian clock regulator | 2.049 | 0.641 | 3.292 |

**Table S6**. Primers used for RT-PCR.

| **Species** | **Gene target** | **Name** | **Sequence 5'-3'** |
| --- | --- | --- | --- |
| *Z. mays* | *ZmPEPC* | HA27-F | CGTCATACAAGCCGGCAATG |
|  |  | HA27-R | TGTGCTTCCAGACTCTGCAG |
|  | *ZmME* | HA29-F | GTCCCAAGATTCGCTCACCA |
|  |  | HA29-R | TGGACACTACTTGCGTGGAC |
|  | *ZmPPDK* | HA30-F | GAATCCCAGAGCATCCCGAG |
|  |  | HA30-R | GTGCAGGACAGGGAAACGTA |
|  | *ZmCA* | HA31-F | AGGTTCTCCAGGGACAGGTT |
|  |  | HA31-R | GGACGGGTTCCACAAGTTCA |
|  | *ZmMDH* | HA40-F | GCCCCTCTCGGCCGC |
|  |  | HA40-R | CACCTTCGAGGGCTTGAAACG |
| *O. sativa* | *elongation factor 1 alpha* | EF1a-2F | TGCCGTGCTCATCATTGACT |
|  |  | EF1a-2R | TTGTCAGGGTTGTAGCCGAC |

**Supporting Data Legends**

**Data S1** (separate file). Isotopomer and metabolite amounts, ^13^C enrichments and relative isotopomer abundances in wild-type (WT) rice and three transgenic lines expressing enzymes of the C_4_ metabolic pathway.

Isotopomer and metabolite amounts (nmol g^-1^ FW). Metabolite amounts were calculated by summing isotopomer amounts. As PEP, 3PGA and pyruvate isotopomers could not be quantified by LC-MS/MS, peak areas for each isotopomer are presented (indicated with ^a^). Their total amounts were determined enzymatically. For better visualisation of the data, samples are grouped into four experiments. Experiment A was performed with WT and transgenic lines (A-1: ^13^CO_2_-pulse labelling; A-2: pulse-chase labelling). Plants were harvested at 60 and 61 DAS (the later one is indicated by asterisks). Note that some samples are common in both groups (i.e. 30 s ^13^CO_2_-pulse/0 s chase). Experiment B was performed only with WT harvested at 79 DAS (B-1: ^13^CO_2_-pulse labelling; B-2: pulse-chase labelling). n.d., not determined.

Relative isotopomer distribution (%) and ^13^C enrichment (%). Values from the 30 s time point are the mean ± SD of 3-4 biological replicates. Values at all other time points are from individual samples or means of two biological replicates. As PEP isotopomers could not be quantified by LC-MS/MS, relative isotopomer distribution and ^13^C enrichment are calculated based on peak areas for these metabolites.

Estimation of ^13^C fluxes. To estimate ^13^C fluxes, fractional ^13^C enrichment for a given metabolite at a given time was multiplied by the number of C atoms in the molecule (*n*) and the metabolite amount using amounts determined in each sample (y = ^13^C enrichment**n**amounts versus time). As malate and aspartate exhibited near-linear accumulation with time (Supplementary Figure 8), slopes were calculated from the entire time kinetic. For malate, two options were used for calculation: using time points from 5 to 600 s or time points from 5 to 300 s as the data for Line B6 at 600 s may be an outlier. For aspartate, time points with no labelling were excluded. For the reminding metabolites, which had non-linear kinetics for ^13^C accumulation (Supplementary Figure 8), a Hanes linear transformation x/y (= time / (^13^C enrichment*amount)) versus x = time was done. Slopes and intercepts were calculated and used to determine Y_max_ (= 1/slope) and X_0.5_ (= intercept/Y_max_) using the equation y = (Y_max_*x)/(X_0.5_+x), y_1_ and y_2_ values were calculated with x_1_= 5 and x_2_= 10, respectively, and the initial slopes (= (y_2_-y_1_)/(x_2_-x_1_)) were determined. No estimation of ^13^C fluxes was made for citrate due to the extremely low labelling of this metabolite.

**Data S2** (separate file). The gene read counts obtained by RNA sequencing for wild type (WT) and transgenic rice lines expressing the enzymes of the C_4_ metabolic pathway. For details of analysis see Materials and Methods.

Supporting Methods

**Method S1. Generation of transgenic rice plants.** Rice calli were induced from mature seeds on N6D medium with 0.4% gellan gum. Calli were inoculated with *A. tumefaciens* carrying the plasmid of interest in 2N6-AS medium supplemented with 20 mg L^-1^ acetosyringone and then co-cultured for 3 days on 2N6-AS medium with 0.4% gellan gum at room temperature in the dark. Afterward calli were washed with sterile water containing 150 mg L^-1^ timentin, blotted with sterile paper towels and placed on N6D medium with 0.4% gellan gum at 25 ᵒC in the dark. thereafter, all media contained 150 mg L^-1^ timentin and either 35 mg L^-1^ hygromycin for EC18089 construct or 3 mg L^-1^ bialaphos for EC18089B construct. Transformed calli were transferred to fresh N6D medium every 10-14 days. After about four weeks of selection, resistant calli were transferred to the regeneration medium RE-III with 0.4% gellan gum and incubated at 24 ᵒC, irradiance of 70 μmol m^−2^ s^−1^ and 8 h light/16 h dark photoperiod; the medium was changed every 10-14 days. Regenerated plantlets were transferred to HF medium with 0.4% gellan gum for rooting and kept in the same conditions as for regeneration. Healthy seedlings were transferred into pots with soil and plants were grown to seed using standard plant growth conditions (see below).

**Method S2. ^13^CO_2_ labelling and quenching procedure.** Labelling chambers were custom-designed and constructed at the University of Western Australia (correspondence about the design and supply of chambers should be addressed to Martha Ludwig). The chambers (shown in Fig. S2) consisted of two Plexiglas plates (12 cm x 14.6 cm x 0.6 cm). The upper plate (lid) had a liquid N_2_ port connected to a plastic funnel via a short poly-vinyl chloride (PVC) tube, which could be stoppered using a removable plastic plug. This port was in the corner of the lid to avoid shading the leaves placed in the chamber. The bottom part of the chamber had an exit port to release liquid N_2_, also fitted with a removable plastic plug. The exit port was placed diagonally opposite the entry port to ensure that the inner chamber was fully flooded with liquid N_2_ when this was poured into the entry port. Two openings in the side of the chamber walls were fitted with metal connectors for attachment of PVC tubing (internal diameter 2 mm), to serve as gas inlet and outlet ports. The internal faces of the two Plexiglas plates were sealed at the edges by soft rubber gaskets, leaving an internal chamber with dimensions of 7.1 cm x 9.8 cm x 1.2 cm (volume 83.5 mL). At a flow rate of 10 L min^-1^, as used during experiments, the gas half-time was 0.35 s. The labelling chamber assembly was held closed by two spring-loaded plastic clamps.

Artificial air mixtures were prepared from separate N_2_, O_2_ and CO_2_ gas cylinders (Air Liquide, Germany) using stationary WMR 4008 gas mixers (Westphal Mess-und Regeltechnik GmbH, Ottobrunn, Germany). One mixer was used to prepare an unlabelled gas mixture containing 79% (v/v) N_2_ and 21% (v/v) O_2_ and 400 ppm ^12^CO_2_. The second was used to prepare a CO_2_-free gas mixture containing 79% (v/v) N_2_ and 21% (v/v) O_2_. The flow rate was 10 L min^-1^ and the gas mixtures were bubbled through purified water at 30°C to obtain a relative humidity of 65%. A GC40 mass flow controller (Brooks Instrument, Hatfield, PA, USA) connected to a 10 L cylinder of ^13^CO_2_ (99.0 atom % ^13^C; Sigma-Aldrich, St. Louis, Missouri, USA) was used to supply ^13^CO_2_ at a flow rate of 4 mL min^-1^. This gas flow was mixed with the humidified CO_2_-free air mixture (flow rate 10 L min^-1^), using a custom-made Tee gas mixer (Bronkhorst, Ruurlo, the Netherlands), to give a final concentration of 400 ppm ^13^CO_2_. During experiments, both unlabelled and ^13^C-labelled gas mixtures were continuously running, directed either to the labelling chamber or to a CO_2_ trap (soda lime) to capture ^12^CO_2_/^13^CO_2_ (Fig. S3). A stainless steel 1-piece 40 series 4-way ball valve (Swagelok, Solon, OH, USA) was used to control which gas mixture entered the labelling chamber. This valve was placed 15 cm away from the chamber to minimize the dead-volume between the valve and chamber.

Labelling experiments were performed between 4-9 h after dawn, when photosynthesis was assumed to be operating at a steady-state, and were performed within the plant growth chamber. The labelling chamber was connected to the gas supply and flushed with the unlabelled air mixture during assembly of the apparatus and positioning of the leaves to be labelled. The first fully expanded leaves (irradiance at leaf level was 450 μmol photons m^−2^ s^−1^) were placed in the labelling chamber without shading and remained attached to the mother plants. To obtain enough material for downstream analysis, two or three leaves were used simultaneously. After placing the leaves in the chamber, the liquid N_2_ entry and exit ports were closed with removable plastic plugs and the chamber assembly was held closed by the spring-loaded clamps. After being placed in the labelling chamber, the leaves were allowed to photosynthesise for 1 min, before switching the gas supply to the chamber to the ^13^C-labelled gas mixture using the 4-way valve. After labelling with ^13^CO_2_, the leaves were rapidly quenched in liquid N_2_. This was achieved by removing the plugs from the entry and exit ports and immediately pouring approximately 300 mL of liquid N_2_ into the entry port, with the liquid nitrogen flowing out through the exit port being collected in a large plastic beaker (Fig. S3). As soon as the plant material was quenched, the ^13^C-labelled gas mixture was re-directed to the CO_2_ trap by switching the 4-way valve. The leaves were detached from the mother plants by cutting and the chamber opened. To avoid thawing of the leaf tissue, liquid N_2_ was regularly poured on the leaf sections while the non-illuminated parts of the leaf, covered by the gasket, were excised and discarded. The ^13^CO_2_-labelled leaf tissue was removed from the chamber and stored at −80 °C.

**Method S3. Immunolocalisation of C_4_ enzymes on leaf sections.** Leaf strips (3 x 10 mm) were collected from the middle portion of the youngest fully expanded leaf and vacuum infiltrated in fixative solution [25 mM sodium phosphate buffer (pH 7.2), 4% (w/w) paraformaldehyde, 0.2% (w/w) glutaraldehyde, 0.01% (w/w) Tween 20] for 15-30 minutes or until tissues sank. Tissues were transferred into fresh fixative solution and incubated for 3-4 hours at 4 ᵒC. After rinsing in 25 mM sodium phosphate buffer (pH 7.2), thin leaf sections were hand-cut using razor blade and placed into blocking solution [20 mM trisaminomethane (pH 7.0), 154 mM NaCl, 0.1% (w/w) Tween 20, 3% (w/v) non-fat milk powder] for 2 hours at room temperature. Sections were incubated in primary antibody in blocking solution (1:100) overnight at 4 ᵒC. The primary antibodies that were used for western blotting were also used for immunolocalisation, except for NADP-ME: since IRRI antibody cross-reacted with Rubisco, we used the antibody prepared by R. Sharwood. Primary antibody was removed by rinsing the sections in blocking solution and was followed by a 2-hour incubation in goat anti-rabbit Alexa Fluor 488-conjugated (Life Technologies, Eugene, OR) or goat anti-mouse Alexa Fluor 488-conjugated (ab150113, Abcam, Cambridge, UK) secondary antibody in blocking solution (1:200) in the dark at 37 ᵒC. After rinsing in blocking solution, sections were treated with 0.05% (v/w) calcofluor white for 5-10 minutes to stain the cell walls.

**Method S4. Leaf gas-exchange analysis.** To assess CO_2_ assimilation rate and stomatal conductance under different atmospheric O_2_ partial pressures (*p*O_2_), a dry CO_2_**-**free airflow rate of 3.9 L min^−1^ with different O_2_ mole fractions (mmol mol^−1^) was used to feed both LI-6800 simultaneously. Five *p*O_2_ in the range from 1.91 to 28.72 kPa (20 to 300 mmol O_2_ mol^-1^ air) were generated by blending the complementary volumetric fractions of N_2_ and O_2_. The two gases were released from two pressurised gas tanks through two mass flow controllers (MKS type 1179, MKS Instruments Inc., Andover, MA) piloted by a Tylan M.F.C. Black box (Tylan General Inc., San Diego, CA). An oxygen sensor (Maxell KE-25F3, Figaro Engineering Inc., Osaka, Japan) was connected in line with the outlet airstream to verify the accurate O_2_ level in the circuit. Leaves were first acclimated to the atmospheric *p*O_2_ of 20 kPa and CO_2_ partial pressure *p*CO_2_ (*C*_a_) of 37 Pa for 20 min. Afterwards leaf gas-exchanges response were determined during stepwise increase of *p*CO_2_ from 0 to 190 Pa each 2-3 min. On the same leaf gas-exchange responses to increasing *p*CO_2_ (0 to 37 Pa) were taken at *p*O_2_ of 9.5, 1.9, 14.3 and 28.6 kPa (100, 20, 150 and 300 mmol O_2_ mol^-1^ air, respectively).
